# Impact of Rapid Molecular Screening at Hospital Admission on Nosocomial Transmission of Methicillin-Resistant *Staphylococcus aureus*: Cluster Randomised Trial

**DOI:** 10.1371/journal.pone.0096310

**Published:** 2014-05-16

**Authors:** Sandrine Roisin, Christine Laurent, Olivier Denis, Michèle Dramaix, Claire Nonhoff, Marie Hallin, Baudouin Byl, Marc J. Struelens

**Affiliations:** 1 Departments of Clinical Microbiology, Erasme Hospital, Université Libre de Bruxelles, Brussels, Belgium; 2 Infection Control, Erasme Hospital, Université Libre de Bruxelles, Brussels, Belgium; 3 School of Public Health, Université Libre de Bruxelles, Brussels, Belgium; Hospital de São Francisco Xavier, CHLO, Faculty of Medical Sciences, New University of Lisbon, Portugal

## Abstract

**Design:**

Cluster randomised crossover trial with seven wards randomly allocated to intervention or control arm.

**Setting:**

Medical and surgical wards of a university hospital with active MRSA control programme.

**Participants:**

All patients hospitalized >48 h in study wards and screened for MRSA on admission and discharge Intervention: Rapid PCR-based screening test for MRSA compared with control screening test by enrichment culture using chromogenic agar.

**Objective:**

We determined the benefit of PCR-detection versus culture-based detection of MRSA colonisation upon patient admission on early implementation of isolation precautions and reduction of hospital transmission of MRSA.

**Main outcome:**

Cumulative rate of MRSA hospital acquisition of in patients screened negative on admission.

**Randomization:**

The sequential order of inclusion of study wards in each arm was randomised by assigning a number to each ward and using a computer generated list of random numbers.

**Findings:**

Of 3704 eligible patients, 67.8% were evaluable for the study. Compared with culture, PCR-screening reduced the median test reporting time from admission from 88 to 11 hours (p<0.001) and the median time from admission to isolation from 96 to 25 hours (p<0.001). MRSA acquisition was detected in 36 patients (3.2%) in the control arm and 34 (3.2%) in the intervention arm. The incidence density rate of hospital acquired MRSA was 2.82 and 2.57/1,000 exposed patient-days in the control and intervention arm, respectively (risk ratio 0.91 (95% confidence interval, 0.60–1.39). Poisson regression model adjusted for colonisation pressure, compliance with hand hygiene and antibiotic use indicated a RR 0.99 (95% CI, 0.69 to 1.44).

**Interpretation:**

Universal PCR screening for MRSA on admission to medical and surgical wards in an endemic setting shortened the time to implement isolation precautions but did not reduce nosocomial acquisition of MRSA.

**Trial registration:**

clinicaltrials.gov NCT00846105

## Introduction

Meticillin-resistant *Staphylococcus aureus* (MRSA) is endemic in acute and chronic healthcare facilities in Belgium as in other European countries [1]. MRSA infections affect more than 170.000 patients annually in the European Union, and have been linked to significant risk of excess morbidity, mortality and healthcare costs [1].

In acute care settings, MRSA colonization places the individual patient at increased risk of healthcare associated infection and constitutes the main reservoir for transmission to other hospitalized patients. Screening admitted patients for MRSA and isolation of MRSA carriers are recommended to prevent MRSA transmission within healthcare facilities [2]–[4]. However, the optimal modalities for detection of MRSA carriers among patients admitted to healthcare facilities, to target patient isolation and decontamination procedures, are still open questions [2]; [3]; [5].

Conventional screening of MRSA is performed by using selective and differential agar media, but the results are not available before 18 to 48 hours [3]. Faster detection can be achieved by using polymerase-chain reaction (PCR) based assays. Among those, the Gene-Xpert system (Cepheid, USA) allows detection of MRSA within 75 minutes from nasal swab samples. This automated system allows random access with immediate processing of each sample when it reaches the laboratory [6].

The medical added value and cost effectiveness of implementing routine testing with molecular assays remains debated. Mathematical modelling suggests that molecular MRSA screening embedded in the “search-and destroy” policy should be highly cost-beneficial at hospital level in both endemic and low prevalence settings [7]. However, conflicting results have been found in intervention studies on the impact of rapid screening on control of MRSA transmission and infection within hospitals [8]–[11]. This heterogeneity of reported effect can be related to variation in the quality of study design, with a majority of studies with positive effect using uncontrolled observational designs, and to differences in the epidemiological and patient care context.

## Objectives

We sought to measure the benefit of rapid PCR versus culture for detection of MRSA carriage upon admission to hospital general wards on (1) shortening the delay to isolate previously unknown MRSA carriers and (2) reducing MRSA transmission to other patients admitted to the same ward. As the hospital transmission of MRSA is primarily occurring by indirect contact through care procedures within a patient care unit, a cluster randomised design was chosen with inclusion of high incidence general wards as study clusters. With such an intervention, randomisation of individual participants would be ineffective due to MRSA cross-contamination within a ward. Practically, only ward randomisation was feasible. As the number of wards that were eligible for the trial was small and to adjust for different transmission dynamics due to case-mix heterogeneity between specialty wards, we have chosen a “cross-over” design. The objectives pertain to both individual level and cluster level. Whereas the delay in implementing patient isolation precautions was assessed at the individual participant level, the primary outcome of hospital acquisition of MRSA was analysed at the cluster level.

## Methods Section

### Patients and Methods

The protocol for this trial and supporting CONSORT checklist are available as supporting information; see Checklist S1 and Protocol S1.

The study protocol was approved by the institutional ethical committee (Ethics committee Erasme hospital) before the study began. All patients admitted to study wards were informed orally and by a written notice explaining the rationale, design, and procedures of the study. The requirement for individual patient informed consent was waived by the ethical committee based on the lack of additional sample collection for the study and provision of standard care to all study participants irrespective of study intervention.

### Trial Design

This observer-blind, cluster randomized intervention trial, used a before-after crossover design with 1∶1 cluster allocation ratio between intervention and standard practice. The trial started in November 2008 as double centre study in two hospitals in Belgium, discontinued for futility after interim data analysis in one hospital and completed as single centre study in January 2010 in the other hospital.

### Participants and Setting

The study initially included two sites, Erasme hospital in Brussels and St Jan hospital in Bruges, Belgium. It was discontinued for futility after five months in the latter site when interim analysis indicated lower than expected MRSA acquisition rate in the control arm.

Erasme hospital is an academic tertiary care centre with 864 beds in 33 wards. The seven wards selected for the study (cardiac surgery, cardiology, pneumology, neurology, oncology geriatric care and rehabilitation wards) had experienced the highest MRSA carriage rates on admission and highest hospital acquisition rates in the year preceding the study and represented a range of specialist medical, surgical and rehabilitation care. Patients expected on admission to study wards to stay more than 48 h were eligible for entry in the study and underwent admission and discharge screening for MRSA carriage. Patients readmitted to study wards more than 24 h after discharge or transfer to a non-study ward were counted as new admissions.

Erasme hospital has a long established MRSA surveillance and control programme in accordance with national guidelines [12]. For more than a decade before the study, periodic alcohol-based hand hygiene promotion campaigns had been successfully conducted hospital wide. MRSA control policies included targeted screening of MRSA carriers among high risk admissions by selective enrichment culture of nasal, throat and wound sites, computer flagging and pre-emptive isolation on readmission of previously known MRSA carriers, isolation of MRSA carriers in single bed room using gloves, gown and mask for patient care and topical decolonisation therapy of MRSA carriers with nasal mupirocin ointment and chlorhexidine bathing. The rate of MRSA hospital acquisition as detected by clinical cultures had been steadily decreasing for 10 consecutive years prior to the study to reach a mean of 1.3 case/1,000 admission in 2008.

The study was conducted in four phases. Phase (1) was a three-month baseline protocol implementation phase to achieve a target of 80% compliance with admission and discharge MRSA screening and isolation of MRSA carriers. Phases (2 and 3) spanned an 11-month intervention phase, subdivided into two five-month intervention periods using either conventional admission MRSA screening test by culture methods performed six days a week during working hours (control arm) or rapid admission MRSA screening performed by PCR testing seven days a week during working hours (intervention arm). The sequential order of inclusion of wards as cluster participants in each study arm was randomised by assigning a number to each ward and using a computer generated list of random numbers. Four wards started with intervention phase for the first five-months and followed with control phase for the following five-months whereas the other three wards followed the reverse sequence. A one-month wash out period (4) without intervention by PCR testing was performed between phase (2) and (3) before crossing the study wards over intervention arms. Evaluable patients had to be screened at admission (or within 24 h) and at discharge (or within 72 h prior to discharge) with a 48 h minimum time interval between these two screenings.

### Intervention

#### Screening process

Within the first 24 h after admission and 72 h prior to the discharge but preferably on the day of discharge, whenever possible, eligible patients were screened for MRSA by swabbing anterior nares, throat, perineum, and wounds, bladder or intravenous catheter exit site if applicable.

Screening swabs of the control arm were processed by conventional testing using selective enrichment culture in 7.5% NaCl Brain-Heart Infusion broth combined with plating on chromID MRSA medium (bioMerieux, France) as previously described [13]. Laboratory technicians processing the swabs for MRSA screening were not aware from which arm of the study patients the samples were obtained, thereby providing single blind of observer determination the major study endpoint. For the intervention arm, admission nasal swabs were splitted and simultaneously processed by both conventional and PCR testing (XpertMRSA assay, Cepheid, USA) as recommended by the manufacturer whereas swabs from other body sites were processed by conventional testing only [13]. Susceptibility testing and identification procedures were detailed elsewhere [13].

The diagnostic accuracy of Xpert MRSA assay was compared with conventional culture on nasal swabs tested during the study period. The sensitivity, specificity, positive and negative predictive value were 60.7, 97.3, 37.8 and 98.9%, respectively, as reported elsewhere [13].

#### Notification of screening results and process time recording

Results of MRSA screening tests were immediately reported through the laboratory information system to the infection control staff and clinical staff in study wards. MRSA positive results were also immediately notified to them by telephone. Study investigators recorded the time of admission to the ward, and time of isolation of MRSA positive patients whereas the time of screening sample reception in the laboratory and time of result notification were recorded in the laboratory information system.

#### MRSA control measures

All patients were cared with standard precautions including alcohol-based hand hygiene. All previously known MRSA carriers and newly detected MRSA positive patients were isolated in an individual room and MRSA contact precautions applied including the use of alcohol-based hand hygiene, gloves, masks and disposable gowns. Topical decolonisation therapy by mupirocin 2% nasal ointment (Bactroban Nasal, GlaxoSmithKline) and chlorhexidine 40 mg/mL soap wash (Hibiscrub, Mölnlycke) was applied during five days to all patients who tested MRSA positive by either culture and/or PCR. Isolation precautions were discontinued after obtaining three consecutive series of negative screening cultures from all previously positive body sites.

### Outcomes and Definitions

The primary outcome was the incidence density rate of nosocomial MRSA acquisition per 1000 patient-days defined as the number of new cases of hospital-acquired MRSA (first positive culture >48 h after admission) divided by the number of patient-days at risk (defined as cumulated lengths of stay by patients who stayed ≥3 days in study wards and were MRSA negative on admission). Secondary endpoints included (1) time between admission and notification of MRSA positive culture/PCR, (2) time between admission of newly detected MRSA carrier and their placement in isolation, (3) The percentage of captured MRSA isolation days defined as the number of patient-days of MRSA positive patients in isolation divided by total number of patient-days of MRSA positive patients [14], (4) cumulative incidence of MRSA nosocomial acquisition per 100 admissions (defined as number of new cases of hospital-acquired MRSA divided by the number of admitted patients who stayed ≥3 days and were MRSA negative on admission by culture and PCR testing X 100) (5) cumulative incidence of MRSA nosocomial infections defined as the number of new cases of hospital-acquired MRSA infection (according to CDC definitions for nosocomial infection and with first MRSA positive culture >48 h after admission) divided by number of admitted patients who stayed ≥3 days in study wards.

### Data Collection

Demographic data on all study participants were recorded from the hospital medical information system. Data were collected at ward level on the following confounding factors: (1) healthcare staff compliance with standard hand hygiene policy and adherence to MRSA contact isolation precautions, as assessed during unannounced ward visits by trained infection control staff member using unobtrusive direct observation, (2) total antibiotic use, measured in cumulated number of Defined Daily Dose (DDD) of J01 ATC class of antimicrobial drugs as defined by the World Health Organisation retrieved from patient level pharmacy records, and (3) MRSA colonisation pressure, as determined by proportion of MRSA colonization-days/total patient-days during the preceding month in each study ward (Table 1).

**Table 1:** Demographic data of study participants, patient care practices and exposure to MRSA carriers in study wards by intervention arm.

Characteristics	Intervention	Control
Study period (months)^a^	11.5	11.5
No. of admissions	3 182	3 251
No. eligible admissions (stay >48 h)	1 788	1 916
No (%) evaluable patients	1 233(68.9)	1 272 (66.4)
No. of patient days at risk	13 233	12 743
Median (range) age (years)	67 (17–101)	69 (15–99)
Men/women	654/579	686/586
Median (range) length of stay (days)	8 (3–182)	8 (3–108)
No. of surgical admissions	236	268
No. of medical admissions	997	1004
No. of admissions by ward		
Pneumology	181	197
Neurology	226	181
Oncology	185	233
Cardiac surgery	234	270
Cardiology	224	195
Geriatric care	113	132
Rehabilitation	70	64
No. of hospital deaths	26	24
No. of patients discharged alive	1207	1248
% MRSA culture positive on admission	13.8	11.8
% MRSA colonisation pressure (MRSA positive patient days/No. patients-days)	7.1 (2521/35745)	7.3 (1975/27257)
Total antibiotic use (DDD/1 000 patients-day)	1 169	1 252
% hand hygiene compliance (No. appropriate/No. observed hand hygiene opportunities)	73.9 (566/766)	63.4 (474/748)
% MRSA patient isolation compliance (No. correct precautions/No. patient observations)	79.8 (103/129)	76.6 (108/141)

a Excluding baseline period.

### Statistical Analysis

Based on MRSA surveillance data from our institution, a 3% cumulated incidence was the expected baseline rate for the primary endpoint. The expected size of effect in our study was based on the hypothesis that shortening time to detection of MRSA carriage by three days would reduce MRSA nosocomial acquisition by 50%. Based on a 2-tailed test sample size calculation for detecting a reduction in proportions from 3% to 1.5% in two independent samples with 95% confidence and a power of 80%, a minimum of 1664 patients were required in each study arm. The study was interrupted at St Jan hospital site after interim analysis of phase 2 showed a MRSA acquisition rate (0.97%) below expected rate in the control arm. The estimated sample size required for achieving 80% power at Erasme hospital was revised to 1 027 patients per arm based on interim analysis of higher than expected MRSA acquisition rate (4.8%) in the control arm in phase 2 of the study. The actual number of 2 505 patients gave the study 70% power to detect a reduction in overall MRSA acquisition rate from 3.2 to 1.6%, not taking into account cluster effect and crossover matched design.

Outcome measures were computed for each ward and month for the intervention and control arms and compared. Given the small number of clusters, analysis of nosocomial MRSA acquisition was based on cluster-level summaries taking into account the crossover design (paired analysis). As described by Hayes & Moulton [15], covariate adjustment was achieved through a two-stage procedure. First, a Poisson regression model, including terms for the matched pairs and for the covariates is fitted to the data in order to obtain the expected number of events and residual for each cluster. Based on these results, in a second step, adjusted rate ratio, confidence interval and significance tests can for this adjusted rate ratio can be obtained. The covariates included the following confounding factors: patient age and sex, monthly number of patient days, monthly MRSA colonisation pressure, staff compliance with hand hygiene, compliance with isolation precautions and total monthly antibiotic use. Participant demographic features, MRSA colonisation pressure and compliance with infection control measures were compared by intervention arm using Chi-square test. Median times from admission to detection of MRSA colonisation and isolation were compared using Wilcoxon rank sum test. Incidence of MRSA infection was compared using Fisher exact test. Analysis was done using STATA version 10.1.

### Cost Data

The screening costs include: tests, laboratory labor, laboratory equipment and labor for taking swabs. The prices of consumables were provided by the manufacturers and the hourly average wage of technician lab/nurse was provided by the Human Resource Department of our institution. Prices are provided excluding VAT. (Table 2).

**Table 2:** Resource use and costs of screening procedures.

Item	Units	Costs^c^ (€)
**Screening**		
Eswab – transport Swab	1	0.65
Take swab by nurse	5 (min)	2.5
Total screening costs		3.15
**Screening - PCR**		
PCR – test cost per sample	1	38
PCR – GeneXpert equipment per sample^a^	1	0.92
Clinical lab. technician time per sample	1.5 (min)	0.75
Total PCR costs		39.67
**Screening - Chromogenic**		
Chromogenic plate	1	1.134
Enrichment broth	1	1.13
Clinical lab. Technician time per sample	5 (min)	2.5
Total chromogenic costs (negative sample)		4.764
Additional costs for a positive sample		
Identification (by coagulase)	1	0.58
Antibiogram (disk diffusion)^b^	1	1.881
Clinical lab. technician time per sample	5 (min)	2.5
Total additional costs (for a positive sample)		4.961

a Costs for Xpert MRSA include: platform costs (price GeneXpert 56000 €), depreciation (5 years) and 8%maintenance costs when 17000 patients per year are screened.

b Antibiogram includes cefoxitine 30 µg and mupirocin 20 µg (Oxoïd, Basingstoke, UK).

c Excluding VAT.

## Results

The study began on 3^rd^ November 2008 and finished on 31^st^ January 2010. During the baseline pre-intervention phase, the target compliance rates with key processes of care were either achieved (admission screening cultures performed in 90% of eligible patients and compliance with infection control precautions 83%) or gradually approached (discharge screening cultures in 68% of eligible patients).

### Participant Flow

**Figure 1:**
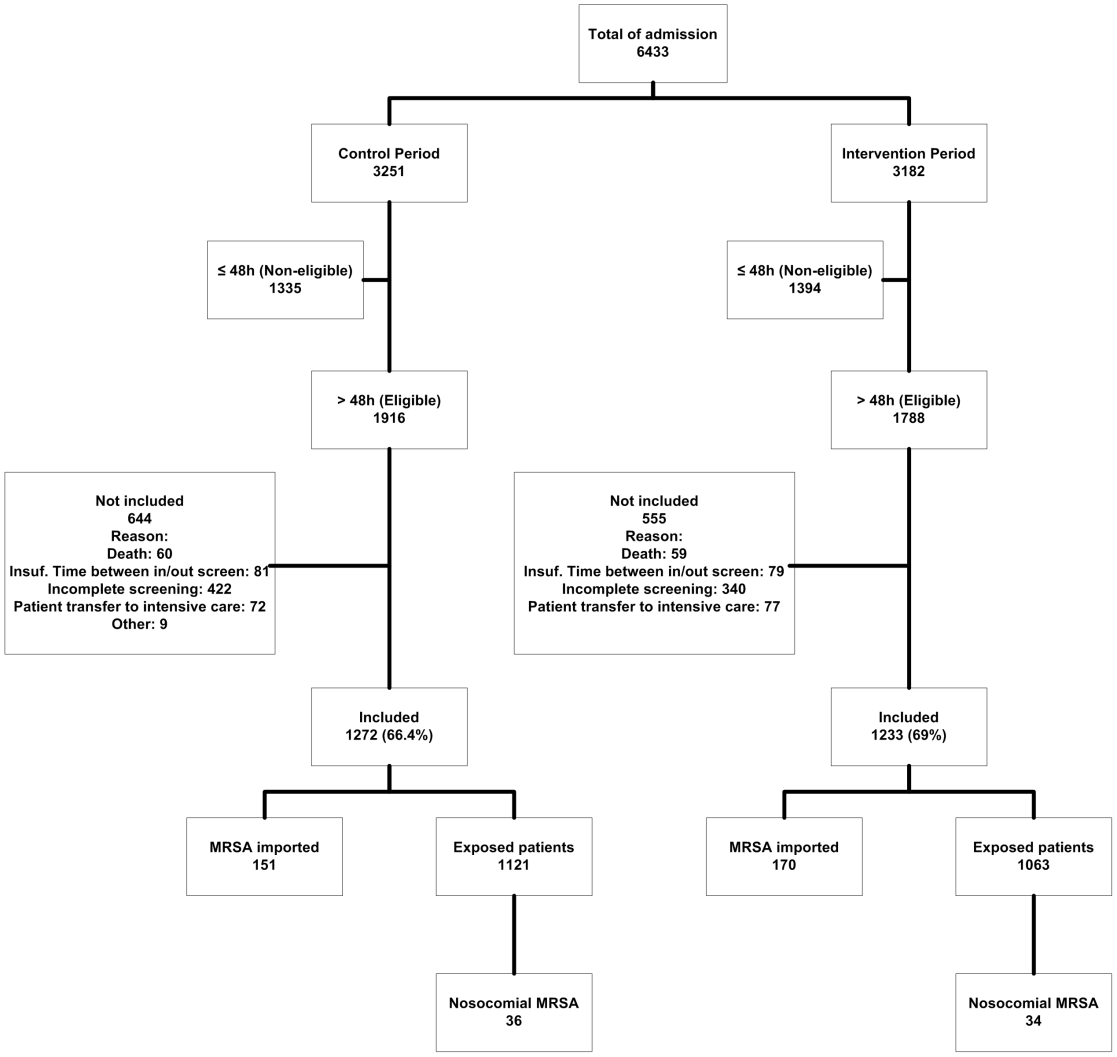
Flow of study patients.

During the study period, a total of 6 433 patient were admitted to study wards. Of 3 704 eligible patients who stayed >48 h, 2 505 (67.8%) patients were evaluable for the study, including 1 233 patients in the intervention arm and 1 272 in the control arm (Figure 1). Demographic data of study participants, patient care practices and exposure to MRSA carriers in study wards are shown by intervention arm in Table 1.

### Outcome

The overall MRSA carriage rate at admission was 12.8% (n = 321). Among these MRSA carriers, 140 (43.6%) patients were newly identified by systematic screening on admission (49 patients in the control arm and 91 patients in the intervention arm) and 181 (56.4%) were previously known MRSA carriers (Table 3).

**Table 3:** Time for MRSA reporting and rate of MRSA acquisition by intervention arm.

Outcome	Intervention	Control	P value
Median reporting time for MRSA admission screening (h)	11	88	<0.001
Median time from admission to isolation of newly detected MRSA carriers (h)	25	96	<0.001
Proportion of isolation days/total MRSA positive patient days (%)			
For newly detected MRSA carriers	82 (943/1147)	73 (528/724)	
For all MRSA carriers	79(1955/2461)	82(1949/2385)	
MRSA acquisition during hospital stay			
No cases/No patients at risk (%)	3.2	3.2	0.986
No cases/1 000 patient-days	2.6	2.8	0.692

**Figure 2:**
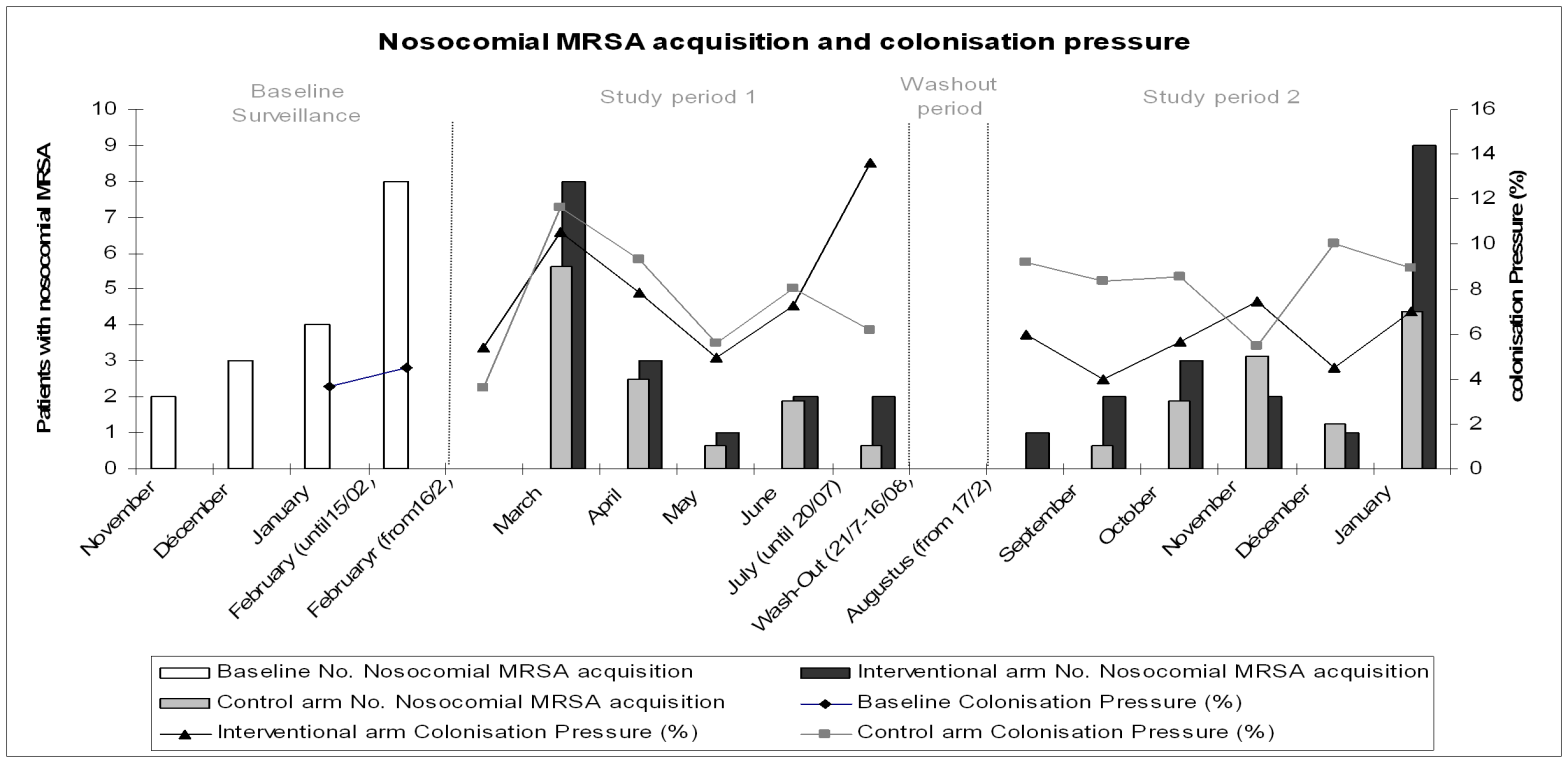
Monthly number of patients who acquired MRSA during their stay and monthly MRSA colonisation pressure by study group and trial phase.

Figure 2 shows the monthly number of patients who acquired MRSA during their stay and monthly MRSA colonisation pressure by study group and trial phase. Overall, 36 patients (3.2%) in the control arm and 34 patients (3.2%) in the intervention arm acquired MRSA during the study period, which correspond to an incidence density rate of nosocomial MRSA acquisition of 2.83 and 2.57/1 000 patient-days (p = 0.69), in the control and PCR arm, respectively, giving a crude risk ratio of 0.91 (95% confidence interval, 0.60–1.39). The adjusted risk ratio for the predefined confounders using Poisson regression model was 0.99 (95% confidence interval, 0.69–1.44) indicating no significant difference in density rate of nosocomial MRSA acquisition between intervention arms.

Compared with culture-based screening, PCR-based screening for MRSA on admission reduced the median turnaround time from admission to notification of positive results from 88 h (range, 39 h 28 min to 6 days) to 11 h (range, 2 h 28 min to 26 h) (p<0.001) and reduced the median time between admission and isolation of newly detected MRSA carriers from 96 h (range, 39 h 35 min to 7 days) to 25 h (range, 2 h 23 min to 7 days) (p<0.001) (Table 3).

Resource utilisation for isolation of MRSA carriers cared in single room with additional precautions was 1.78-fold higher in the intervention arm compared with control arm, with use of 943 versus 528 isolation-days, respectively.

The percentage of captured MRSA isolation days for newly detected MRSA carriers was 73% in the control arm and 82% in the intervention arm, suggesting marginal improvement by rapid PCR screening [14]. However, the overall percentage of captured MRSA isolation days for all MRSA carriers, including newly detected MRSA carriers, previously known MRSA carriers and hospital-acquired MRSA carriers was not modified, 81% and 80% in the control and the interventional arm, respectively (Table 3).

A low incidence rate of nosocomial MRSA infection was observed during the study: only seven cases of MRSA infection occurred, two in the control arm and five in the intervention arm, or a cumulative incidence of 1.57 and 4.06 infection/1 000 patients respectively, p value = 0.281).

## Discussion

This cluster-randomized, cross-over controlled trial did not identify reduction of nosocomial MRSA acquisition by using PCR based universal screening for MRSA carriage at admission to high risk medical and surgical wards in a teaching hospital, despite a fourfold shorter delay between admission and isolation of newly detected MRSA carriers using PCR versus culture based screening. Previous studies of this intervention have yielded conflicting results. Quasi-experimental studies using historical controls suggested effectiveness of rapid PCR based MRSA screening in preventing MRSA acquisition or infection in surgical, intensive care or general acute care hospital settings [5]; [6]; [8]; [9]; [11]. A prospective, cross-over study of PCR based MRSA screening showed a decrease in MRSA transmission in surgical wards attributed to enhanced compliance with decolonisation therapy [16]. However, two large randomised controlled trials and a meta-analysis showed no measurable benefit of PCR over culture for screening in general wards or in planned surgery patients [17]–[19]. Another study showed no benefit of universal over targeted PCR-based MRSA screening [20]. The heterogeneity of effect observed across studies may be related to variation in many factors, including study design, patient population, MRSA prevalence and transmission rates, diagnostic accuracy and turnaround time of PCR and culture methods, compliance with standard precautions, isolation, decolonisation and antibiotic stewardship [1]; [2].

There are several hypotheses to explain the lack of impact of rapid screening in our study. Firstly, in contrast to studies showing a marked effect of introducing carrier screening and isolation as the sole MRSA control measure in hospitals with uncontrolled endemic transmission, our institution had been conducting a multimodal intervention programme including antibiotic stewardship, hand hygiene promotion and active MRSA surveillance and control measures for over 15 years as recommended by national guidelines [12] and hand hygiene promotion campaigns [21]. This infection control program is well implemented in our hospital, as indicated by staff compliance with hand hygiene policy and isolation precautions observed in this study. As part of this baseline control programme, over half of MRSA positive patients at admission to the study hospital were pre-emptively isolated based on computer flagging of history of MRSA colonisation or infection. Secondly, the PCR assay we used had a low sensitivity in our study population. However, the true admission MRSA prevalence was not underestimated due to this low PCR sensitivity as we controlled screening with optimized culture methods performed in parallel with PCR. Despite a marginal increase in proportion of captured isolation days for newly detected MRSA carriers, overall captured MRSA isolation days did not differ. Thus, in our setting, rapid MRSA screening did not enhance the high baseline compliance with isolation and decolonisation procedures.

In addition, use of PCR screening may result in untoward effects. In our hospital with a relatively low prevalence of MRSA, the PCR assay, despite an acceptable specificity, presented a low positive predictive value when compared to culture [13]. Therefore, the number of isolation days doubled in the intervention arm due to many patients that were reported MRSA positive by PCR but not confirmed by culture. A study examining the clinical and epidemiological significance of only PCR-positive patients found no increased risk of infection compared to PCR-negative patients [22]. Undesirable consequences of over-isolation cannot be ignored as this measure carries a substantial cost burden, increased workload for hospital staff and risk of decreased quality and safety of patient care [23].

Our study has several limitations. Firstly, only patients staying more than 48 hours were included in the study and therefore shorter stay patients who could serve as MRSA donors were not investigated. Secondly, the cluster-randomised design that assumes no transmission between clusters was to some degree compromised by contact of patients admitted to different wards of the hospital, as transfer of patients and staff between wards occurred during the study. Thirdly, while our PCR screen had the shortest turnaround time reported to date, by providing results within admission day but often during the night shift, it actually took longer to start isolation of patients after reception of test result. This delay is not uncommonly observed in other clinical settings [6], [11], [17]. Patient care processes and diagnostic processes need to fully align to optimise benefit from rapid diagnostic tests.

In our setting, as costs for PCR testing were at least 4 times higher than by conventional method, and as PCR did not demonstrate any advantage in terms of MRSA transmission, rapid testing by PCR was considered not cost-effective. A PCR-based screening may be effective in other settings. In low MRSA prevalence countries where pre-emptive isolation policy apply to high risk patients admitted to hospital, MRSA screening can help reduce the number of unnecessary pre-emptive isolation days [24]. However, only culture-based screening by use of chromogenic agar, and not PCR screening, was found to be cost saving [25]; [26].

In conclusion, in a hospital with an active culture-based MRSA surveillance program and good adherence to standard and isolation precautions, the use of a rapid PCR-based system for screening MRSA carriers at admission showed no measurable effect in enhancing infection control measures or reducing MRSA transmission. Decision about use of appropriate technology for MRSA admission screening must take into account the determinants of infection control effectiveness related to the hospital context.

## Supporting Information

Protocol S1 **Complete protocol for this trial.**
(DOC)

Checklist S1 **CONSORT checklist for cluster randomized trial.**
(DOCX)

File S1 **Ethics approval document.**
(PDF)
