# Supplementary material for: Impact of Rapid Molecular Screening at Hospital Admission on Nosocomial Transmission of Methicillin-Resistant Staphylococcus aureus: Cluster Randomised Trial
Source: PLoS One. 2014 May 16;9(5):e96310. doi: 10.1371/journal.pone.0096310 (PMC4023928; doi:10.1371/journal.pone.0096310)
Supplement: Protocol S1 — Complete protocol for this trial. (DOC) [file pone.0096310.s001.doc]

# Study reference- Erasme-ULB- P2008/201

# NIH register NCT00846105 (ClinicalTrials.gov)

# Final Protocol-Version 10- 15.12.2008;

# Addenda 7.2.2009; 24.4.2009

# Two-Center Intervention Study to Evaluate the Impact of Rapid Molecular Screening on Nosocomial Transmission of Methicillin-Resistant *Staphylococcus aureus* (MRSA).

# Brief title: Efficacy study of rapid test to prevent hospital transmission of MRSA

# *Investigators:*

# Marc Struelens*, Baudouin Byl*, Olivier Denis*,

# Christine Laurent*, Marie Hallin*, Michèle Dramaix**

# Bart Gordts***, Eric Nulens***

# Departments of Microbiology and Infection Control,

# ULB-Hopital Erasme*, and Department of Statistics, ULB-School of Public Health**, Bruxelles

# and AZ StJan***, Brugge

## Sponsors:

## Federal Public Service Public Health, Food Safety and Environment, Belgium.

## Cepheid, Europe.

## Introduction

Methicillin-resistant *Staphylococcus aureus* (MRSA) strains have become endemic pathogens in acute and chronic healthcare facilities in Belgium and are also spreading in the community. MRSA infection is causing increased public concern as it carries a significant risk of morbidity, mortality and has been linked to substantial excess healthcare costs (1). The increased risk of prolonged hospital stay and fatal outcome from bloodstream or surgical infection with MRSA, as compared with no infection or infection with methicillin-susceptible *S.aureus*, has been well documented (1).

Efficient control of MRSA transmission within healthcare facilities critically depends on screening for and isolation of MRSA carriers among admitted patients. Active surveillance cultures for MRSA are now part of clinical practice recommendations both in Europe and the USA (2-3). Indeed, studies have indicated that up to 70 % of the patient reservoir for MRSA among hospitalized patients can only be detected by active sampling and selective cultures of muco-cutaneous colonization sites, including the nares, throat, skin, wounds, rectum and insertion sites for invasive devices. MRSA colonization places the individual patient at increased risk of healthcare associated infection and constitutes a reservoir for transmission to other hospitalized patients. There is an urgent public health need for early and reliable detection of carriers of MRSA among patients admitted to healthcare facilities, to inform patient isolation and decontamination procedures, and thereby more effectively control cross-infection (4).

Detection of MRSA in muco-cutaneous swab specimens is typically performed by using selective and differential agar media, sometimes enhanced by enrichment broth culture which is incubated overnight before plating on selective agar (5). Traditional media contain an indicator system to presumptively identify *S.aureus*, such as mannitol and pH indicator like phenol red combined with inhibitory agents such as high NaCl concentration or antimicrobial agents. These media usually require 48 to 72 h before presumptive identification can be made, followed by confirmation on isolated colonies by additional testing which require up to 24 h (5). New selective agar media supplemented with cefoxitin and chromogenic enzyme substrates have shown improved performance, with some media allowing MRSA identification with > 80 % sensitivity and 99 % specificity after 18-24 h and with 95-100 % sensitivity and > 90 % specificity after 42-48 h (5).

New molecular assays for MRSA detection are undergoing clinical and economic evaluation. The first generation tests were based on multiplex PCR detection of two targets, the resistance determinant *mec*A and *S.aureus* specific genes (*nuc, fem*A, *coa*). Although these assays are sensitive enough for rapid (< 4h) detection in broth cultures from blood or swab specimens, they suffer from limited sensitivity and poor specificity in specimens with mixed flora containing methicillin-resistant coagulase-negative staphylococci (CoNS) and methicillin-sensitive *S.aureus* (MSSA*)*. Current real-time PCR assays are more sensitive with an analytical detection level in the range of 10 genome copies. A commercial RT-PCR test, the IDI-MRSA (GeneOhm Sciences- Becton Dickinson) is designed to target MRSA-specific DNA elements that bridge the SCC*mec*-chromosomal junction (*orfX*)(6). This design prevents false-positive signal to occur from mixed flora specimens containing MSSA and MR-CoNS. Such co-colonization is found in approximately 3 to 5% of nasal swab samples from hospitalized patients. This PCR assay is, however, potentially affected by *mecA* deletion from SCC*mec* elements (false-positive signal from MSSA strains) and divergent sequence variants in SCC*mec* (false-negative signal from MRSA strains). Validation studies on international *S.aureus* strain collections and clinical samples indicate that these atypical strains are rare (7). Prospective clinical evaluations of this assay have shown that MRSA could be detected from nasal swabs in 2h with high sensitivity (> 93 %) and specificity (>98 %) and somewhat lower sensitivity with skin and throta swab specimens (12;13). Real-time multiplex PCR on the LightCycler system (Roche) has also shown very promising performance with testing nasal swabs from neonates and adult patients (8). Another promising in house RT-PCR assay used an immuno-magnetic separation step for selective MRSA concentration prior to DNA extraction and amplification (9) Very recently, a fully automated RT-PCR system, the Gene-Xpert system (Cepheid, USA) has been developed for rapid detection of MRSA within 75 minutes. This system is very attractive because it requires minimal sample preparation and allows random access with immediate processing of each sample when it reaches the laboratory. The diagnostic accuracy of the Xpert MRSA system is currently under clinical evaluation in the USA and Europe. Preliminary results indicate sensitivity of 98.5 % and specificity 97% (Mehta M et al; Cepheid, data on file).

Because of the increased cost of implementing routine high volume testing with these molecular assays, demonstration must be made of their added medical value and cost effectiveness for facilities fighting healthcare-associated MRSA infection. Likewise, the many studies that document the impact of rapid MRSA carrier screening on outbreak management and control of MRSA transmission within hospitals are subject to some degree of bias, such as uncontrolled/unplanned before-after design. Evidence from such intervention studies and from mathematical modeling suggests that enhanced rapidity and accuracy of molecular MRSA screening embedded in the “search-and destroy” policy should be highly cost-beneficial at hospital level in both endemic and low prevalence settings (10).

Five intervention studies using historical controls and assessing multiple interventions suggested effectiveness of rapid MRSA screening in surgical, intensive care or general acute care hospital settings (11-15). However, two recently published, large, well designed randomised controlled trials failed to confirm such a benefit in general wards or in planned surgery patients (16, 17).

Additional well-designed intervention trials are needed to clarify the medical and financial added-value of these strategies in diverse healthcare systems with different levels of MRSA endemicity and infection control policies. These require multidisciplinary technology assessment and system-wide practice change to be met. The present proposal attempts to contribute to this evaluation of medical benefit of PCR-based detection of MRSA carriers on reducing the risk of in-hospital transmission of MRSA in the Belgian healthcare setting.

## Study objectives

The general objectives of this intervention study to be conducted in parallel in two large Belgian hospitals are to measure the benefit of rapid (< 3 h) PCR detection of MRSA carriage upon admission on shortening the delay to implement contact isolation precautions for carriers and reducing of nosocomial MRSA transmission.

**Primary aim:**

Primary outcome: To determine if a ≥ 50 % reduction of incidence of nosocomial MRSA acquisition can be observed after replacing culture by PCR for universal MRSA screening of patients upon admission to high incidence units in two acute care hospitals

**Secondary aims:**

To compare the following secondary outcomes during periods of MRSA carrier screening among hospital admissions by means of rapid PCR testing versus conventional culture:

1. Median time required for detection of MRSA carriage after admission
2. Median time required for starting isolation of MRSA carriers
3. Number of patient-days of MRSA carrier stay in non-isolated conditions
4. MRSA nosocomial infection rate
5. MRSA cross-transmission rate
6. Sensitivity and specificity of PCR vs conventional MRSA screening

## Study design

**Study type and patient population**

The study will be an open label, **cluster randomized intervention trial,** with **cross-over design.** It will be carried out in parallel over a one year period in two large hospitals in

1. patients admitted to high risk surgical, medical and geriatric wards of Erasme hospital (7 to 8 wards)
2. patients admitted to the intensive care departments, medical and geriatric wards of AZ St Jan hospital (6 to 7 ward).

The Erasme hospital is an 858-beds academic hospital located in Brussels, including medical and surgical care departments. The AZ-St Jan hospital is an 800-bed general teaching hospital located in Brugge, including medical and surgical care departments. Both institutions have an integrated MRSA surveillance and control program in place for over 10 years. These programs adhere to the national recommendations for MRSA control in acute care hospitals (BICS-CSH/HGR 2003). These include: active screening of MRSA carriers on admission by selective culture of nasal, throat and wound sites in high risk patients and/or units, flagging of previously detected MRSA carrier patients at re-admission, placement of MRSA carriers in single room with contact isolation precautions and decolonisation therapy of carriers with nasal mupirocin and antiseptic bathing for 5 days.

The choice of a minimum size of effect of 50% reduction in MRSA acquisition is based on the assumption that a 6-fold reduction of transmission rate can be expected from placing MRSA positive patients in isolation, and that the reduction from 3 to 0 days of unisolated stay can be expected by the intervention. Assuming a baseline rate of 3% nosocomial acquisition rate in the exposed population of study wards, the study population will include 3,700 patients in each hospital with approximately 50 new cases of MRSA acquisition in the control arm (n= 1,850 patients) versus 25 new cases in the intervention arm (n= 1,850 patients).

Selection of study wards in each hospital will be defined based on determination f wards with highest MRSA nosocomial incidence documented by local surveillance data for the pre-trial period first half of 2007, to meet the expected number of new cases per 5-month period above. Because discharge surveillance is not currently performed in the study centres, it is assumed that adding pre-discharge screening of MRSA carriers will increase the detection rate two-fold and double the actual (3%) versus retrospectively observed incidence (1.5 %). This assumption will be checked during the 3-month baseline screening reinforcement phase. Any type of adult care ward will be considered for inclusion in the study, including intensive care, medical, surgical and geriatric wards.

**Intervention study design**

The study will be a **cluster randomized intervention trial,** with **cross-over design** carried out in three phases:

(1) a 3 month screening reinforcement phase during which the adherence to the admission and discharge MRSA screening protocols will be promoted and monitored monthly to achieve > 80 % compliance rate, application of isolation precautions to MRSA colonized patients will be measured and checked to achieve > 80 % compliance and the MRSA acquisition rate will be checked to lie within 95 % confidence limits of the projected 3% estimate. In addition, the length of stay per ward will be measured and checked for homogeneous distributions.

(2) a 5 month conventional detection test (standard care) phase during which MRSA screening will be performed by optimized conventional culture methods 6 days a week during working hours.

(3) a 5 month rapid detection test intervention phase during which admission MRSA screening will be performed by rapid PCR testing 7 days a week during working hours.

After eligible wards in each hospital have been checked to meet these inclusion criteria in phase (1), the sequential order of inclusion of eligible study wards in phase (2) and (3) will be randomly assigned within each centre by assigning a number to each ward and using a computer generated list of random numbers per study arm in each hospital so that one half of the wards will start phase (2) for the first 5 months and then phase (3) for the following 5 months whereas the other study wards will be “crossed-over” to follow the reverse sequence. No wash-out period will be used between phase (2) and (3). If the analysis of patient length of stay by ward indicates homogeneous distribution with markedly different median values between wards, stratified randomisation of wards within short/long (below/above pooled median) stay groups will be considered.

The planned study start date is: November 3^rd^, 2008 for the baseline observation and completion is planned on December 31^st^, 2009 (post-poned to end January, 2010 see addendum).

Admission screening: evaluation of patient sampling procedures:

During the entire study period (baseline and intervention phase), the infection control nurse responsible for the study will perform daily rounds in each of the study wards to ensure that screening of all newly admitted patients is being done systematically and without delay.

In addition, during a defined period, he or she will collect individual patient data on a subset of 20 % of included patients to identify risk factors for MRSA carriage based on history taking (notion of previously recorded MRSA carriage, transfer from other department or hospital, transfer from a nursing home, hospital stay in the previous year, attending outpatient care at a clinic in the previous year, presence of chronic skin sores, or living/working in a livestock farm, working as veterinarian or in a slaughterhouse). These data will be analysed to estimate the predictive value of these risk factors for the carriage of MRSA.

In the control arm, as per routine infection control policy in each institution, all patients admitted to study wards and presenting with will be sampled within 24 h after admission by taking swabs of anterior nares, throat, perineum and of any wounds, bladder catheter or intravenous catheter exit site (see appendix 1 for conventional MRSA screening procedures). Patients with previously recorded active MRSA carriage will be presumptively placed in contact isolation until three new series of conventional selective cultures of relevant body sites are found negative.

In the rapid test intervention arm, all patients admitted to study wards will be sampled within 24 h after admission. To ensure comparison of like with like, sample taking will include: (1) a swab from the anterior nares for PCR testing according to the test manufacturer’s instructions (see appendix 2 for rapid PCR screening test procedure); (2) the swab of anterior nares, and swabs from throat, perineum and of any wounds, bladder catheter or intravenous catheter exit site will be processed by conventional testing (see appendix 1 for conventional MRSA screening procedures). Patients with previously recorded active MRSA carriage will not be sampled for PCR testing but placed in pre-emptive isolation until three series of conventional selective cultures of relevant body sites are found negative.

Thus, patients in each arm will be managed identically, except that the rapid PCR test will be performed and its results made immediately available to infection control and clinical staff in the intervention arm.

MRSA isolates will be tested for mupirocin susceptibility and stored at -80°C for genotyping.

Additional contact isolation precautions.

MRSA contact isolation precautions will be applied to hospitalised patients as per local policy (see appendix 3 for isolation procedures in HE and AZSJ hospitals) either when previously recorded in the laboratory information file as active MRSA carrier or as soon as newly detected by either culture or PCR. Patients will be released from isolation after three consecutive culture screens for MRSA are negative after the end of therapy. Topical decolonisation therapy will be applied as per routine protocol in each institution (see appendix 4 for decolonisation procedures in HE and AZSJ hospitals) to all patients with either a positive MRSA culture and/or PCR.

Notification of screening results and process time recording.

Results of MRSA screening tests will be notified electronically to clinicians by either the technologist or biologist on duty through the laboratory information system (LIS) as well as with a telephone call to the Infection Control study nurse (during working hours) or the unit head nurse (outwith working hours). For culture tests, both presumptive positive results based on colony pigmentation typical for MRSA on chromogenic selective culture medium as well confirmed identification results will be notified immediately by the same communication modes. The precise timing (date, hour, min) of all telephonic result notifications will be recorded in the LIS.

The date and time (hour; min) of patient admission to study ward, as well as of patient placement in contact isolation or release from isolation will be recorded by the infection control study nurse. The date and time (hour; min) of screening sample reception in the laboratory, as well as of result electronic notification will be recorded by the LIS.

Primary outcome monitoring and case finding procedures.

To monitor MRSA acquisition for primary endpoint measurement, active discharge surveillance will be performed in all patients who have stayed in study wards for more than 48 h by collecting surveillance cultures for MRSA either on the day of being discharged or within 72 h prior to discharge from the study ward. Discharge surveillance sampling will be performed by the study protocol nurse on daily ward rounds during week days, by collecting swabs of nares, throat, perineum, and any wound, bladder or intravenous catheter exit site and endotracheal aspirate in ventilated patients for conventional selective MRSA culture (see appendix 1). For the denominator, a patient will be counted as two ward admissions if staying outside the ward for more than 24 h.

All isolates will be saved at -80°C for typing of relevant isolates to identify transmission clusters. Genotyping will be performed by *spa* sequence analysis and SCC*mec* typing (see appendix 5 for MRSA typing procedure).

Interim analysis and contingency plan.

After the first 10 weeks of the 3-month baseline period, 2 weeks will be used to analyse data and check ward eligibility based on fulfillment of the following “pass criteria”:

(1) > 80 % compliance with admission and discharge conventional culture screening, calculated for weeks 8-10 for the pooled admissions to all study wards;

(2) > 80 % compliance with additional MRSA contact isolation procedures, calculated for weeks 8-10 based on a sample of observations of 50 patient care contacts per hospital in all study wards;

(3) pooled incidence of nosocomial MRSA acquisition ≥ 3 new cases /100 at risk admissions in the study wards for weeks 1-10.

These criteria must be met for each hospital to ensure sufficient compliance with the protocol, a sufficient denominator of exposed number of patients per month in the study wards and target incidence figures. If either of these pass criteria is not fulfilled, the two trial phases can be extended to 6 or 7 months and/or further compliance reinforcement will be undertaken before starting the randomisation of units for inclusion in the intervention phase.

In addition, MRSA isolates will be typed to ensure that no atypical SCC*mec* variants are emerging.

In case of outbreak of MRSA or other infections detected at any time during the study, additional contingency outbreak control interventions may be decided by the hospital’s Infection Control Committee. These non-protocol interventions, if any, will be recorded, described in the final report and taken into account at analysis stage.

**Study endpoints and statistical analysis**

The **primary endpoint** of the study will be the following microbiologically documented outcome:

1. **Incidence density rate of MRSA nosocomial colonisation**, defined as the number of new cases of hospital-acquired MRSA (first positive culture > 48 h after admission) divided by the number of patient days at risk (defined as cumulated lengths of stay by patients who stayed ≥ 3 days and were MRSA negative on admission screening by conventional culture, and, if performed, PCR test).

**Secondary endpoints**

These will include three related process of care indicators:

1. Time (h) between admission and notification of MRSA positive culture/PCR
2. Time (h) between admission and placement of newly detected MRSA carrier in contact isolation

Secondary endpoints will also include the following microbiologically-documented outcomes:

1. Number of patient-days of MRSA positive patients cared for with standard precautions and not isolated
2. Cumulative incidence of MRSA nosocomial colonisation, defined as number of new cases of hospital-acquired MRSA (first positive culture >48 h after admission) divided by the number of admitted patients who stayed ≥ 3 days and were MRSA negative on admission by culture and PCR testing, if performed.
3. Cumulative incidence of MRSA nosocomial infection, defined as number of new cases of hospital acquired MRSA infection (according to CDC definitions for nosocomial infection and with first MRSA positive culture > 48 h after admission) divided by number of admitted patients who stayed ≥ 3 days.
4. Number of clusters of ≥ 2 cases/unit of acquired MRSA colonisation with a clonally related strain, defined as a MRSA strain with the same spa type or PFGE major type.
5. Sensitivity and specificity of rapid PCR test compared to conventional culture for MRSA screening, taking into account one parallel set of admission nasal screening samples per patient.

The **primary analysis** will be based on Poisson regression two level model (individual patient and unit exposure) performed at each hospital level to assess the impact of the intervention on local incidence-density of MRSA nosocomial colonisation, and ajusted for four potentially confounding factors. The dependent variable will be endpoint 1 above. The ward level **independent variables** will include (1) **intervention arm**, (2) MRSA **colonisation pressure** (as determined by monthly proportion of MRSA colonisation-days/total patient-days among patients admitted during the preceding month in one particular ward), (3) **staff** **compliance with standard and isolation precautions** (as determined by a sample of 50 observations per ward/ trial period, including 25 care opportunities for patients under MRSA isolation precautions and 25 for patients cared for with standard precautions; see appendix 6 for precautions compliance monitoring procedure) (4) **antibiotic use** (as determined by cumulated number of Defined Daily Doses of J01 ATC class of antimicrobial drugs per monthly period) and (5) monthly bed occupancy rate.

Heterogeneity of effect by ward/hospital will be assessed. **To quantify the protective efficacy of the intervention** for the at risk population per centre, the adjusted Rate Ratio (and 95% confidence interval) of nosocomial MRSA acquisition will be calculated for the intervention arm in all study wards per hospital. Pooled analysis of average effect for both hospitals will be calculated. In case of significant heterogeneity between hospitals, hospital level will be added as covariate in the model.

**Secondary analyses** will include:

A. Logistic regression two level models (individual patient and unit exposure) will be performed at each hospital level to assess the impact of the intervention on local cumulated incidence of MRSA nosocomial colonisation. The dependent variable will be endpoint 6 above. The **independent variables** will include patient age, sex and the 5 independent ward level indicators as described above for the primary Poisson analysis.

Heterogeneity of effect by ward/hospital will be assessed. Pooled analysis of average effect for both hospitals will be calculated. In case of significant heterogeneity between hospitals, hospital level will be added as covariate in the model.

C. The median process time intervals (endpoints 2-4 above) and number of missed isolation days (endpoint 5 above) will be compared by Wilcoxon tests for patients within the same unit/hospital between arms; median times (interquartile range) will be compared for each study phase and hospital.

D. In addition, monthly rates of endpoints 1, 6 and 7 will be plotted over time and interrupted time-series, segmented linear regression analysis of temporal trends per study periods will be performed for each hospital. The hypothesis to be tested is that there will be a significant drop in the level of rates from the beginning of the intervention compared to the baseline or control period, possibly associated with a negative change of slope due to a decreasing trend in the intervention period.

E. Diagnostic accuracy (clinical sensitivity and specificity) will be calculated for the rapid PCR test (MRSA GeneXpert, Cepheid) compared to conventional culture as gold standard based on all matched nasal swabs (N=3,700) tested in parallel during the 10 month trial period.

**Sample size estimate.**

Based on the cumulated incidence endpoint and study hypothesis that shortening time to detection of MRSA carriage by 3 days will reduce MRSA nosocomial acquisition by 50%, a standard sample size calculation for detecting a difference in proportion between 3% and 1.5% in two independent samples with α=0.05 and ß=0.20 (80 % power) suggests that 1,237 patients should be included in each study arm. Assuming non-independence of samples due to the time dependence of MRSA transmission rates between study phases and taking into account the cluster design, a conservative 150 % sample size estimate would suggest enrollment of 1850 patients per arm in each hospital, or a total inclusion of 7,400 patients.

**Ethical considerations.**

This study protocol (version 8) has been approved by the ethical review committees of both study hospitals in accordance with the international and national legislation on human experimental studies.

Protocol amendments made between the approved version 8 and the final version 10 include: (1) extension of duration of the baseline observation period from 2 to 3 months to allow a pre-trial data analysis step; (2) extension of admission screening to all admissions to study wards, irrespective of presence of individual risk factors, based on the pre-trial finding that up to 85 % of patients admitted to candidate study wards do present with risk factors, making targeted screening essentially irrelevant; (3) clarification of wording for the process/sample size of monitoring the compliance of HCWs with infection control measures and (4) clarification of the determination of patient denominator.

All patients admitted to study wards will be informed orally and by a written notice explaining the rationale, design, and procedures of the study. All patient related data will be strictly protected and accessible only to infection control and laboratory professionals in accordance with legal data protection standards. These data will be centralised and anonymised by the study coordinator in Erasme hospital prior to submission to the statistical department, Ecole de Santé Publique, ULB for statistical analysis.

The requirement for individual patient informed consent has been waived by the ethical committees based on the motivations that: (1) this group intervention diagnostic study cannot be conducted without including 100% patients, (2) the same standard care will be provided to all study participants irrespective of the diagnostic test intervention, (3) lack of patient-care related risk associated with the intervention because no additional invasive procedure or collection of diagnostic sample will be required for the study.

**Acknowledgements.**

The investigators thank Dr Ben Cooper, Health Protection Agency, London, UK for critical review of and helpful comments on the study protocol.

# Two-Center Intervention Study to Evaluate the Impact of Rapid Molecular Screening on Nosocomial Transmission of Methicillin-Resistant *Staphylococcus aureus* (MRSA).

# Addendum 1 to the protocol – modification of analysis plan (February 7^th^, 2009)

Based on the results of the base-line period (November 3^rd^, 2008 –January 15th, 2009), 7 wards of Erasme hospital and 6 wards of AZ St Jan hospital have been randomized and allocated either to the intervention or control arm for the first 5 month study period to begin on February 9^th^, 2009. Stratified randomization of wards was performed according to the hospital and average patients’ length of hospital stay.

In the base-line period, the compliance rates with key processes of care were achieved (admission screening cultures and infection control precautions > 80%) or closely approached (discharge screening cultures 68% and improving during the baseline weeks). However, the incidence of MRSA nosocomial acquisition was lower than hypothesized in the protocol: 0.79% (95%CI: 0.34% - 1.55%). This incidence rate was unexpectedly low compared to previously recorded rates. When considering the higher limit of the 95%CI, the planned sample size allows detection of a 50% reduction in incidence of nosocomial acquisition with 80% power and taking into account a potential cluster effect only if the pooled incidence in both hospitals combined is analysed as the primary endpoint. Therefore, although heterogeneity between the two hospitals will be studied as planned, the pooled results for both hospitals will be presented and analysed as the primary endpoint.

An additional interim analysis is planned three months after the beginning of the study to obtain a new estimate of the incidence of MRSA nosocomial acquisition in the control units and to check if the number of subjects is large enough to reach the objectives.

**Addendum 2 to the protocol – April 23, 2009.**

A one-month wash-out period without intervention by PCR testing being performed in any ward will be followed after the end of phase 1 and before crossing over the sudy wards into phase 2 of intervention.

**Budget V4: to be adjusted for number of PCR tests needed**

| **Personnel** | **N** | **Cost/unit** | **Cost in €** |
| --- | --- | --- | --- |
| Infection control nurse | 24 MM | 50,000/yr | 100,000 |
| Laboratory technologist | 4 MM | 45,000/yr | 15,000 |
| Microbiologist | 2 MM | 70,000/yr | 11,500 |
| **Working costs** |  |  |  |
| Xpert PCR System | 1 | 50,000 | -* |
| Xpert-MRSA test | 1300 to 1850 | 48 | -* |
| Surveillance cultures |  |  |  |
| Admission | 7,400 | 4 | -** |
| Hospital stay/discharge | 15,000 | 4 | 60,000 |
| Molecular typing MRSA | 200 | 15 | 3,000 |
| Data analysis |  |  | 5,000 |
| **Total** |  |  | 194,500 |
| Overhead 15 % |  |  | 29,175 |
| **Grand total (2 hospitals)** |  |  | **223,675** |

* the estimated number of PCR tests is based on the proportion (35 to 50 %) of admitted study patients fulfilling the screening test indication criteria. Test kits (n=600) are to be provided free of charge by Cepheid. Additional kits to be purchased.

** charged to hospital budget (routine MRSA surveillance cultures)

#####

Reference List

(1) Cosgrove SE, Qi Y, Kaye KS, Harbarth S, Karchmer AW, Carmeli Y. The impact of methicillin resistance in Staphylococcus aureus bacteremia on patient outcomes: mortality, length of stay, and hospital charges. Infect Control Hosp Epidemiol 2005; 26(2):166-174.

(2) Muto CA, Jernigan JA, Ostrowsky BE, Richet HM, Jarvis WR, Boyce JM et al. SHEA guideline for preventing nosocomial transmission of multidrug-resistant strains of Staphylococcus aureus and enterococcus. Infect Control Hosp Epidemiol 2003; 24(5):362-386.

(3) Verbrugh HA. Value of screening and isolation for control of methicillin-resistant Staphylococcus aureus. Clin Infect Dis 2005; 41(2):268-269.

(4) Struelens MJ. Rapid identification of methicillin-resistant Staphylococcus aureus (MRSA) and patient management. Clin Microbiol Infect 2006; 12 Suppl 9:23-26.

(5) Brown DF, Edwards DI, Hawkey PM, Morrison D, Ridgway GL, Towner KJ et al. Guidelines for the laboratory diagnosis and susceptibility testing of methicillin-resistant Staphylococcus aureus (MRSA). J Antimicrob Chemother 2005; 56(6):1000-1018.

(6) Huletsky A, Lebel P, Picard FJ, Bernier M, Gagnon M, Boucher N et al. Identification of methicillin-resistant Staphylococcus aureus carriage in less than 1 hour during a hospital surveillance program. Clin Infect Dis 2005; 40(7):976-981.

(7) Warren DK, Liao RS, Merz LR, Eveland M, Dunne WM, Jr. Detection of methicillin-resistant Staphylococcus aureus directly from nasal swab specimens by a real-time PCR assay. J Clin Microbiol 2004; 42(12):5578-5581.

(8) Paule SM, Pasquariello AC, Hacek DM, Fisher AG, Thomson RB, Jr., Kaul KL et al. Direct detection of Staphylococcus aureus from adult and neonate nasal swab specimens using real-time polymerase chain reaction. J Mol Diagn 2004; 6(3):191-196.

(9) Francois P, Pittet D, Bento M, Pepey B, Vaudaux P, Lew D et al. Rapid detection of methicillin-resistant Staphylococcus aureus directly from sterile or nonsterile clinical samples by a new molecular assay. J Clin Microbiol 2003; 41(1):254-260.

(10) Wernitz MH, Swidsinski S, Weist K, Sohr D, Witte W, Franke KP et al. Effectiveness of a hospital-wide selective screening programme for methicillin-resistant Staphylococcus aureus (MRSA) carriers at hospital admission to prevent hospital-acquired MRSA infections. Clin Microbiol Infect 2005; 11(6):457-465.

(11) Harbarth S, Masuet-Aumatell C, Schrenzel J, Francois P, Akakpo C, Renzi G et al. Evaluation of rapid screening and pre-emptive contact isolation for detecting and controlling methicillin-resistant Staphylococcus aureus in critical cre: an interventional cohort study. Crit Care 10, 25. 2006.

(12) Cunningham R, Jenks P, Northwood J, Wallis M, Ferguson S, Hunt S. Effect on MRSA transmission of rapid PCR testing of patients admitted to critical care.J Hosp Infect. 2007 Jan;65(1):24-8.

(13) Conterno LO, Shymanski J, Ramotar K, Toye B, van Walraven C, Coyle D, Roth VR.Real-time polymerase chain reaction detection of methicillin-resistant Staphylococcus aureus: impact on nosocomial transmission and costs.Infect Control Hosp Epidemiol. 2007 Oct;28(10):1134-41.

(14) Robicsek A, Beaumont JL, Paule SM, Hacek DM, Thomson RB Jr, Kaul KL, King P, Peterson LR. Universal surveillance for methicillin-resistant Staphylococcus aureus in 3 affiliated hospitals. Ann Intern Med. 2008 Mar 18;148(6):409-18.

(15) Keshtgar MR, Khalili A, Coen PG, Carder C, Macrae B, Jeanes A, Folan P, Baker D, Wren M, Wilson AP. Impact of rapid molecular screening for meticillin-resistant Staphylococcus aureus in surgical wards. Br J Surg. 2008 Mar;95(3):381-6.

(16) Harbarth S, Fankhauser C, Schrenzel J, Christenson J, Gervaz P, Bandiera-Clerc C, Renzi G, Vernaz N, Sax H, Pittet D. Universal screening for methicillin-resistant Staphylococcus aureus at hospital admission and nosocomial infection in surgical patients. JAMA. 2008 Mar 12;299(10):1149-57.

(17) Jeyaratnam D, Whitty CJ, Phillips K, Liu D, Orezzi C, Ajoku U, French GL. Impact of rapid screening tests on acquisition of meticillin resistant Staphylococcus aureus: cluster randomised crossover trial. BMJ. 2008 Apr 26;336(7650):927-30.

**Appendix 1 A: MRSA admission screening**

**A. Sampling procedure**

**Nasal swab for PCR (GeneXpert MRSA) and conventional MRSA culture:**

Use the specific double COPAN swab to sample both nostrils with the same double swab. Insert the swab in one anterior nostril and rotate gently against the nasal mucosa with a rotating movement then repeat in the other nostril. Insert the double swab in the container and humidify by pushing it in the sponge in the container.

**Nares and other body sites for conventional MRSA culture**

Use a separate swab (conventional COPAN swab in Amies transport medium for aerobic culture) per site for conventional selective MRSA culture:

**Nares**: Perform the same procedure was above for nasal swab.

**Throat** : swab in the back of the throat.

**Perineum:** Strike the swab over the gluteal cleft from scrotum/vagina to anus over perineal area.

**Wounds**: Different wounds under the same dressing can be sampled with the same swab.

**Sputum / bronchic aspirate / urine / wound**

standard procedure of specimen collection of aerobic culture in sterile container.

Complete the laboratory order with patient identification, date and time, requested analysis and send to the microbiology lab. Record sampling in nursing file.

## B. Conventional MRSA screening culture and identification

Samples for MRSA screening in hospitalized patients include swabs from nares, throat, perineum, any skin lesions (decubitus ulcer, insertion site of vascular catheter, …) and urinary meatus if bladder catheter present. Semi-solid Amies transport medium (Copan) will be used.

**Culture**

- Screening swabs will be first plated onto chromogenic selective MRSA agar (ChromID-MRSA, bioMérieux France or ID-MRSA, Bio-Rad, France) and then inoculated into enrichment Brain Heart Infusion (BHI) broth supplemented with 3.5% NaCl.
- After 24 h incubation, broth will be sub-cultured onto MRSA agar.
- Selective agars will be incubated for 48 h at 35°C and inspected daily.

**Identification and oxacillin susceptibility testing**

- Suspected MRSA colonies (green on ChromID-MRSA or red on ID-MRSA) will be identified by coagulase tube test (human plasma).
- Oxacillin susceptibility will be tested by Vitek 2 automated system (bioMérieux, France).
- Preliminary identification of suspected MRSA isolates found in newly detected MRSA carriers will be reported to the Infection Control team by phone. A preliminary identification will be encoded in the Laboratory Information System (LIS).
- Final identification will be reported in LIS as soon as the result of susceptibility testing is available.

**Conservation of isolates**

One MRSA isolate per patient will be conserved at – 80°C for further characterization/genotyping.

**APPENDIX 2 : LABORATORIUM KLINISCHE BIOLOGIE**

**Dienst Microbiologie**

| **Xpert MRSA test** | | |
| --- | --- | --- |
| Opgesteld door: Patrick Descheemaeker |  | |
| Beoordeeld door: Moleculaire bioloog, Verantwoordelijke Moleculaire biologie | | |
| Goedgekeurd door: Stafleden | | |
| Versie: 0 - Geldig vanaf:  Afgedrukt op: 29/04/2011 03:43:00 PM | | Sop Code: MMBMDT00025  Aantal pagina’s: 33 |

# onderwerp

Deze SOP beschrijft een methode voor snelle detectie van MRSA a.d.h. van de moleculair diagnostische Xpert MRSA test.

# toepassingsgebied

De Cepheid Xpert MRSA test, uitgevoerd op het GeneXpert Dx System, is een kwalitatieve in vitro diagnostische test ontworpen voor het snel detecteren van methicilline resistente *Staphylococcus aureus* (MRSA) uit neus-keel-perineum swabs afgenomen bij risico patiënten. De Xpert MRSA test is bedoeld als hulpmiddel in de preventie en het onder controle houden van MRSA infecties in de gezondheidszorg.

# definities en afkortingen

MRSA: methicilline resistente *Staphylococcus aureus*

SPC: Sample-Processing Control

PCC: Probe Check Control

DNA: Deoxyribonucleïnezuur

NA: Not applicable

# principe

Het GeneXpert Dx System automatiseert de Xpert MRSA test door zowel staalopzuivering, amplificatie van DNA en detectie van het doelwit sequentie in één cartridge te integreren. Het systeem bestaat uit een instrument, een PC en software. Het systeem vereist het gebruik van GeneXpert cartridges voor eenmalig gebruik. In de cartridge gebeurt het volledige PCR proces.

# staal

## Staaltype

| Voorkeur | Copan dubbele wisser |
| --- | --- |
| Toegelaten |  |
| Verboden | Katoen wissers, wissers in een gel-matrix |

## Benodigde hoeveelheid

1 wisser (bevat een dubbele swab)

## Transport

Zie SOP “Staalafname, - behandeling en transport” (GALAXX00021)

## Behandeling en bewaarcondities

De stalen kunnen gedurende 24 uur bij kamertemperatuur worden bewaard. Indien langer dan 24 uur moeten de stalen in de koelkast worden bewaard. Stalen die in de koelkast worden bewaard zijn stabiel gedurende 5 dagen.

## Bijzondere voorwaarden

# Veiligheid

Er moet gewerkt worden volgens de geldende richtlijnen van de op het laboratorium gehanteerde algemene veiligheidsvoorschriften (zie SOP GALAXX00007). De veiligheidsvoorschriften voor het gebruik van toestellen en reagentia zijn beschreven in hun respectievelijke SOPs.

# Toestellen

| Toestel | Inventarisnummer/SOP |
| --- | --- |
| GeneXpert Dx System | **MMBTAP00010** |
|  |  |

# hulpmiddelen

| Hulpmiddel | Inventarisnummer/SOP |
| --- | --- |
| vortex | **MALHMH00011** |
| Transfert pipetten | **MALHPI00005** |

# reagentia

| Reagens | Inventarisnummer/SOP |
| --- | --- |
| Xpert MRSA kit | **MMBRKI00039** |
|  |  |

# Kalibratie

## Kalibratiemateriaal

| Standaard | Inventarisnummer/SOP | Frequentie |
| --- | --- | --- |
| NVT |  |  |
|  |  |  |

## Uitvoering

# kwaliteitscontrole

| Controle | Inventarisnummer/SOP | Frequentie |
| --- | --- | --- |
| Nihil |  |  |
|  |  |  |

# werkwijze

## Frequentie en tijdstip van uitvoering

Dagelijks

## Voorbereiding

Haal per staal een”cartridge”en een “set of reagents”uit de Xpert MRSA kit

## Uitvoering

### Genereren van etiketten

Per wisser worden 2 barcodes gegenereerd via Molis hoofdmenu: gebruikersmenu – scanacties – B – X – Screening – accepteren.

(één voor de S tube, één voor de cartridges)

### Vullen van de cartridge

- Haal de cartridge uit de verpakking (raak de reactietube NIET aan!!!)
- Haal de reagentia uit de verpakking (elutiebuffer “s”, reagens “1” en reagens “2”)
- Kleef een barcode op de cartridge
- Kleef een barcode op het glazen flesje met “Elutie Reagens” (zwart deksel)
- Open het glazen flesje met “Elutie Reagens” (zwart deksel)
- Maak één swab los uit het rode deksel mbv een proper watje. Breng de resterende swab terug in de transporttube
- Breng de swab in reagens **“S”** en breek af, maak gebruik van een steriel watje om contaminatie te vermijden.
- Sluit het flesje reagens **“S”** met swab, vortex bij hoge snelheid gedurende minstens10 seconden
- Open terug het flesje en breng met een steriel transfertpipet de volledige inhoud in de “S” (sample) kamer positie van de cartridge **(GEEN SCHUIM OVERBRENGEN!!)**


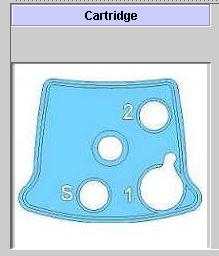


- Breng vervolgens de volledige inhoud van reagens 1 in Kamer 1 van de cartridge
- Breng de volledige inhoud van reagens 2 in Kamer 2 van de cartridge
- Sluit het deksel van de Cartridge

***Opmerking****: De test moet gestart worden binnen de 15 minuten na het vullen van de cartridge*

### Starten van de testprocedure

- Dubbel klik het GeneXpert Dx icoon


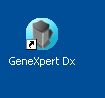


- Log in met je eigen user name en paswoord


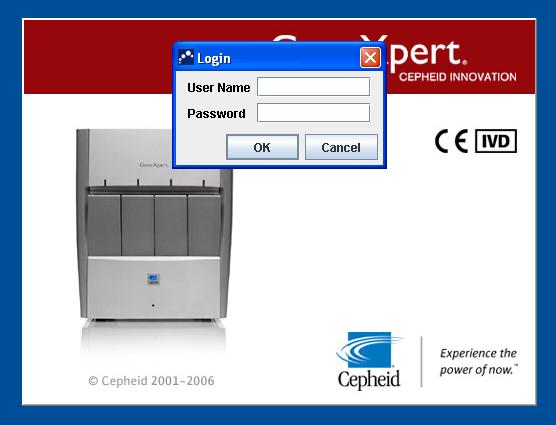


- Na het inloggen verschijnt het volgende scherm


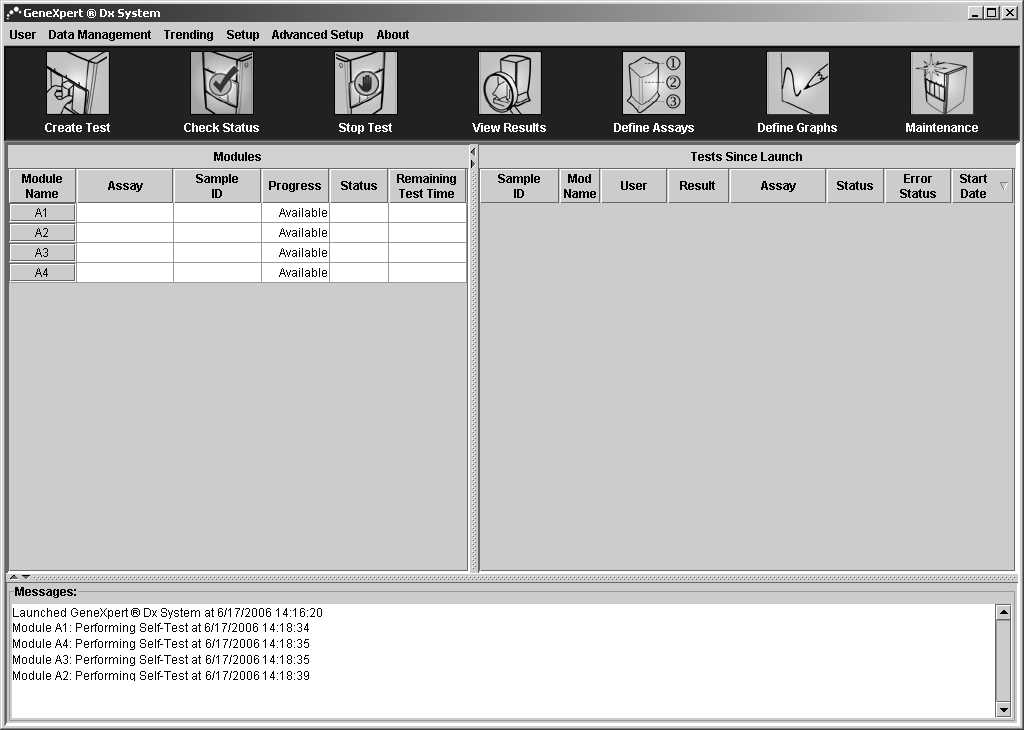


- Klik op het icoon “create test”


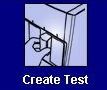


- Het “Scan Cartridge Barcode” dialoogvenster verschijnt


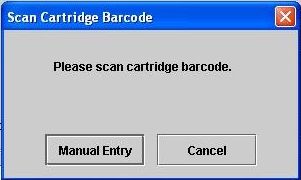


- Neem de gevulde Cartridge en scan de 2D barcode

(Alle gegevens van de cartridge worden in het systeem geladen: testprotocol, lotnummers en vervaldata)

- Na het inscannen van de Cartridge barcode verschijnt het volgende “Scan Sample ID Barcode” dialoogvenster


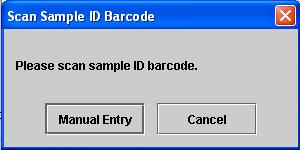


- Scan het labonummer barcode die op de cartridge gekleefd is
- Klik “start test”
- Typ opnieuw je paswoord in
- Open de module van het instrument onder het groene knipperend lichtje
- Breng in deze positie de cartridge in het instrument
- Sluit de deur van de module (de test start en het groen lichtje stopt met knipperen, na het beëindigen van de test dooft het lichtje

## Bewaring – archivering

Nihil

## Afvalverwerking

Zie SOP “Afvalverwerking” (GALAXX00016)

Gooi de gebruikte cartridges in de gele biohazard bak

# Interferenties

# Verwerking van de resultaten

## Berekeningen

NVT

## Meetbereik

Detectielimiet: 50 CFU/swap

## Referentiewaarden

| Omstandigheden | Referentie |
| --- | --- |
| NVT |  |

## Interpretatie

## Alert- en doorbelwaarden

| Omstandigheden | Actie |
| --- | --- |
| Positieve test | 1. tijdens de diensturen   Mailen naar [ziekenhuishygiene.VPK@azbrugge.be](mailto:ziekenhuishygiene.VPK@azbrugge.be)   1. buiten de diensturen   Mailen naar [ziekenhuishygiene.VPK@azbrugge.be](mailto:ziekenhuishygiene.VPK@azbrugge.be) en doorbellen naar de verpleegeenheid. |
|  |  |

Volgende gegevens moeten gemeld worden:

Patiënt <naam> met <patiëntennummer> van <VE> is drager van een MRSA.

Bij het doorbellen van de resultaten vermeld in het LIS bij “telefonische opmerking”, welke persoon verwittigd werd.

## Resultaatverwerking, technische validatie, rapportering

### Interpretatie

Volgende resultaten kunnen bekomen worden:

| Scherm | Interpretatie | Op protocol |
| --- | --- | --- |
| 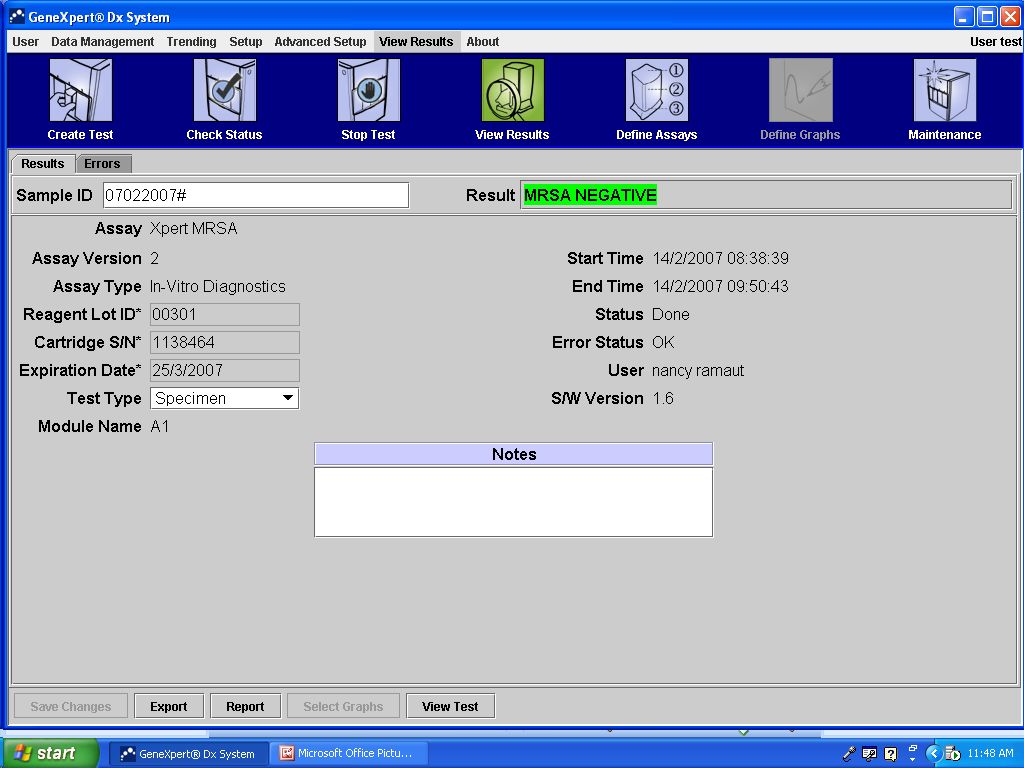 | Negatief | Real-time PCR toont geen MRSA aan. |
| 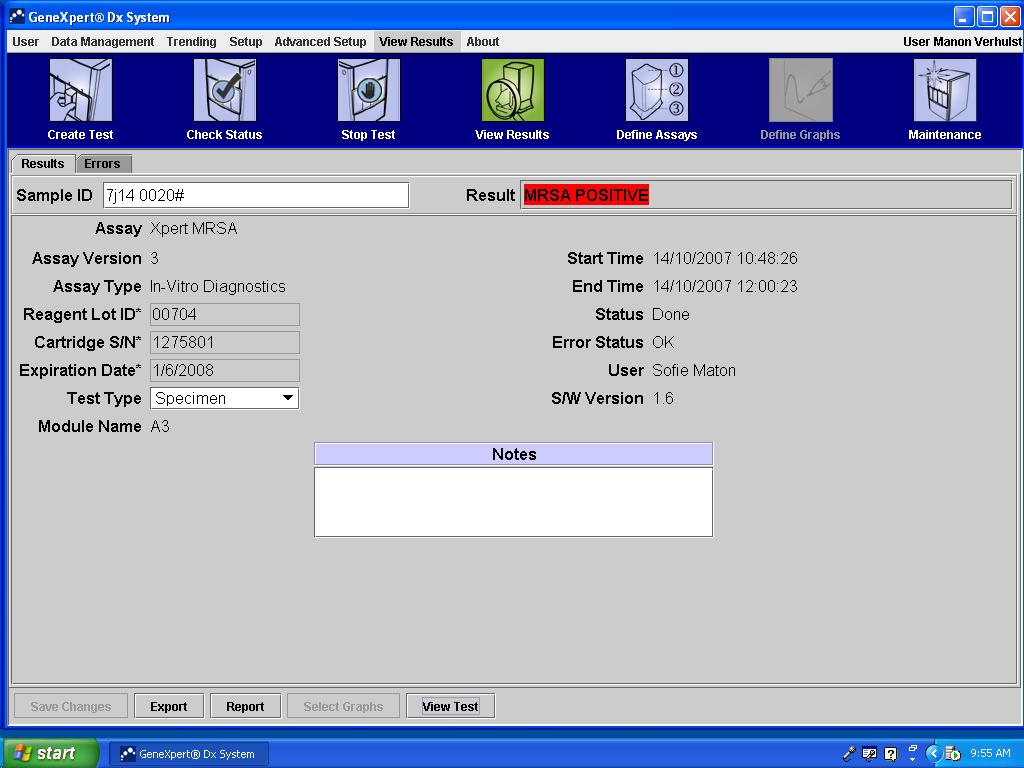 | Positief | Real-time PCR toont de aanwezigheid van MRSA aan.Een ziekenhuishygiënist zal de afdeling contacteren om bijkomende voorzorgsmaatregelen te bespreken |
| 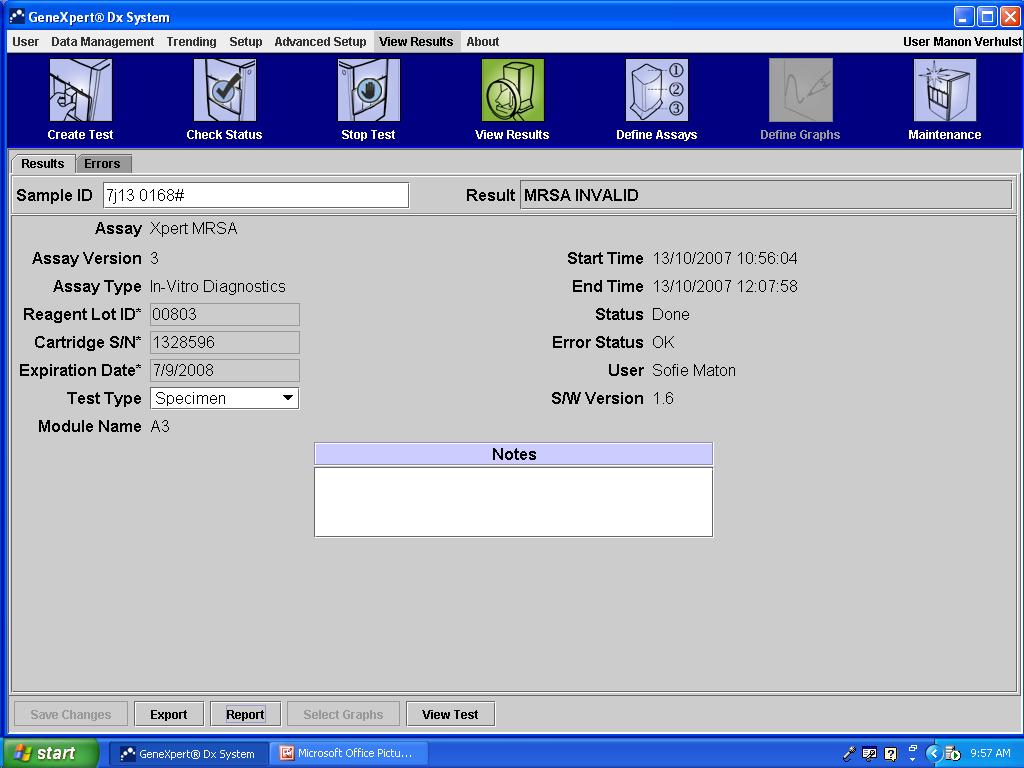 | Inhibitie | inhibitie |
| 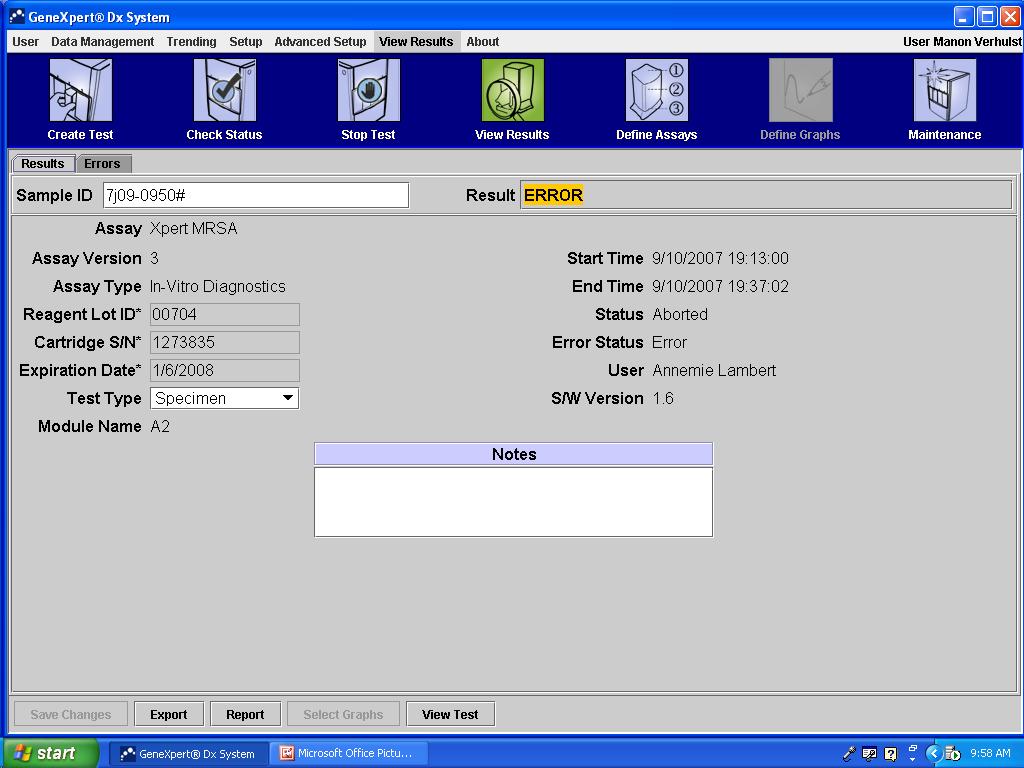 | Error | inhibitie |
|  |  |  |

### Resultaatverwerking

- User – Login

Geef username en paswoord in

- Klik op het icoontje “check status”


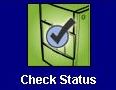


- In de lijst ‘**tests since launch’**: dubbelklik op het gewenste labonummer


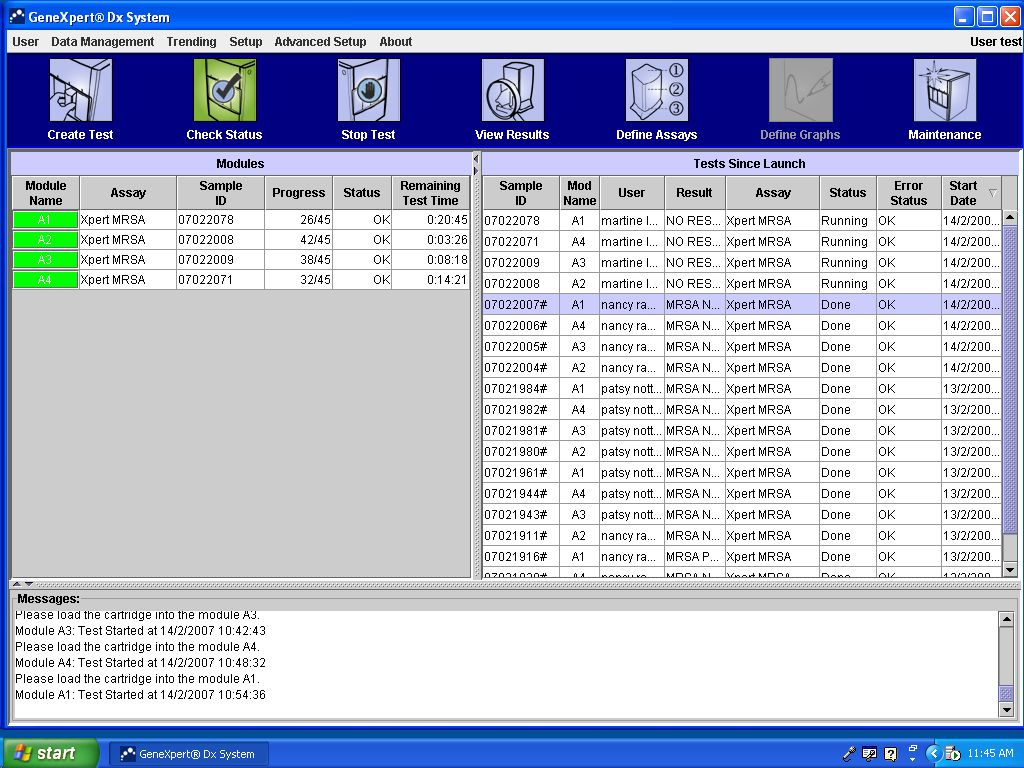


- Het volgende scherm verschijnt


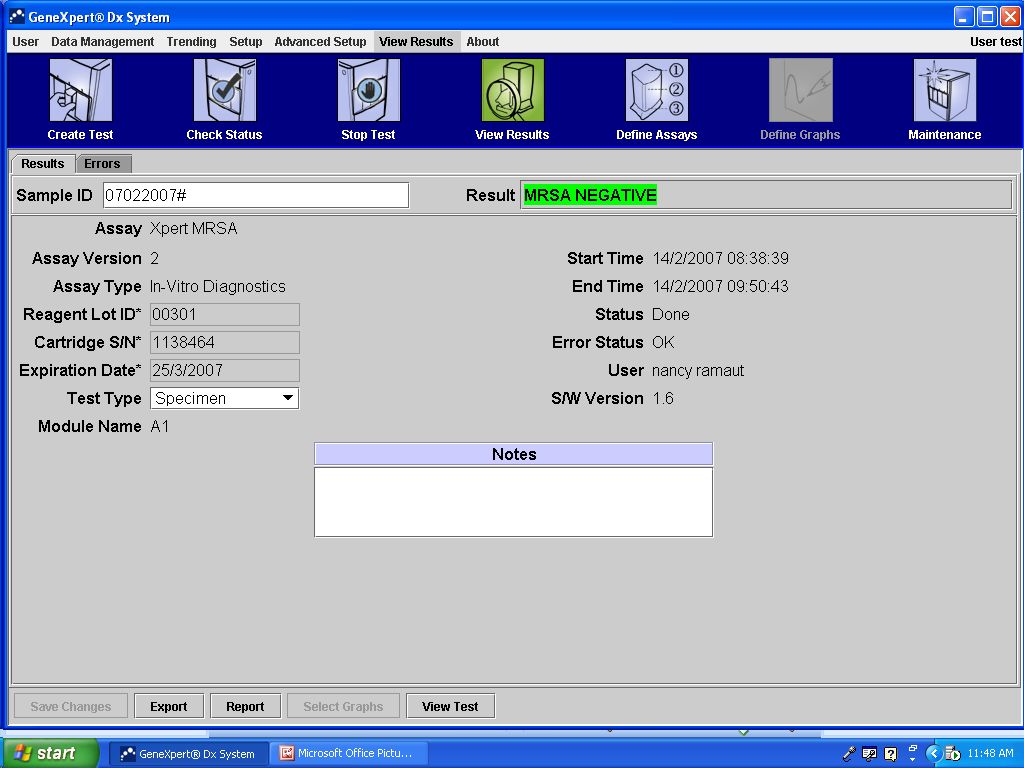


- Vul het sample ID aan met “#” (Shift #)
- Haal de respectievelijke cartridge uit het toestel en schrijf het resultaat erop
- Klik “save changes” en vervolgens “yes”
- Ga eventueel terug naar “check status” om de volgende test te valideren
- User – logout

### Rapportering

- Roep het labonummer in het LIS op via werklijst MRSAKW.
- Beantwoord de test (negatief, positief of inhibitie)

## Aandachtspunten

# opmerkingen

# verwijzingen

| Nummer | LOG/MAP nummer | Titel |
| --- | --- | --- |
| MMBDEX00001 | VIV | Bijsluiter Xpert MRSA |
|  |  |  |

**Appendix 3**: **MRSA colonised patient : Additional MRSA isolation precautions**

Upon receipt of the lab result indicating positive MRSA status of a patient, the attending ward-nurse:

- Initiates ‘contact’ precaution procedure and the decontamination scheme.
- Requests full MRSA screening for the other patient(s) if the patient shares the room and transfers the MRSA patient into a single room.
- Labels the patients’ file with the small sticker ‘contact’
- Places the sign ” MRSA Isolation Precautions” on the patient’s door
- MRSA carriers are nursed in an individual room if available. If not, MRSA carriers can be grouped (cohorted) in a common room.
- Healthcare workers wear gloves and gowns when entering the room if contact is anticipated with the patient or the environment or during medical procedures or transfer out of the room.
- Healthcare workers wear mask when performing procedures that can potentially generate aerosols (e.g. tracheal or bronchial aspiration, dressing of infected wounds, bed sheet change
- The room is cleaned and disinfected daily

The infection control nurse :

- Checks if the patient is placed in ‘contact’ precautions and decontamination has been initiated
- Schedules further screening with the treating physician(s).
- After 3 successive negative cultures the additional “contact precautions” will be stopped.

**Appendix 4. MRSA decolonisation procedure**

**Erasme Hospital:**

***Protocol A:***

Indication: nasal carriage with mupirocin susceptible MRSA (or low level resistance) or limited carriage (no chronic sore, urinary catheter, gastrostomy, COPD) AND no more than one failure of decolonisation

- mupirocin (Bactroban nasal ® ) nasal ointment tid in each nostril during 5 days
- chlorexhidine soap (Hibiscrub ®) shower or wash during 5 days

***Protocol B:***

Indication: all other cases (with a maximum of 3 courses)

- Iso-Betadine dermicum ® soap shower or wash during 5 days
- Iso-Betadine gel ® nose ointment tid in each nostril during 5 days

NB: in case of failure of both protocols: no more attempt.

**Screening after MRSA decontamination:**

Nose, throat, perineum, sputum in case of productive cough, urine in case of urethral catheter, open wounds and entry points. Performed at least 2 days after stopping active antibiotic or antiseptic therapy.

## St Jan hospital

## Nose

Treatment with mupirocin nose ointment (Bactroban®) 3x/ day, during 5 days. In consent with the bacteriologist an other anti-staphylococcus ointment can be used. Administer a small amount of ointment (size of a matchstick head) in the front of both nostrils. Spread the ointment by means of external pressure to the nose.

The treatment must be stopped immediately in case of irritation or swelling of the mucous membranes.

## Throat

Treatment with povidone iodine (Iso-Betadine® mouth wash) 3x/ day during 5 days. Preferably gargle or rinse, if not possible do mouth wash.

## Skin

Wash the entire body of the patient or let the patient wash him-/herself 1x/ day, preferably take a shower, with Iso-Betadine® soap during 5 days. See to it that the entire body surface is soaped and preferably scrubbed well and respect the 1 minute contact time. Wash the patient’s hair once or twice with Iso-Betadine® soap.

Stop treatment immediately in case of irritation.

## Ruptured or damaged skin

Disinfecting ointment, preferably Iso-Betadine® dermicum and Iso-Betadine® gel 2x/ day during 10 days.

If antibiotic ointment must be used, preferably use fucidin or neobacitracin products. Use mupirocin skin ointment (Bactroban®) only as an alternative in order prevent the emergence of mupirocin-resistance.

Stop treatment immediately in case of irritation.

# Screening after MRSA decontamination.

Nose, throat, perineum, sputum in case of productive cough, urine in case of urethral catheter, open wounds and entry points.

Note “MRSA” on every microbiology application form for every site.

Screen only if the patient is not getting any active antibiotics or antiseptics.

The patient must be antibiotic and antiseptic free for at least 2 days.

Appendix 5 : MRSA typing by *spa* sequence analysis

For *spa* amplification, the PuRe Taq Ready-To-Go PCR Beads (Amersham Biosciences, Freiburg, Germany) are used in a total volume of 25 µl containing LiChrosolv water (Merck, Darmstadt, Germany), DNA, and 10 pmol of each HPLC-cleaned primer spa-1113f (5'- TAA AGA CGA TCC TTC GGT GAG C -3') and spa-1514r (5'- CAG CAG TAG TGC CGT TTG CTT -3').

Thermal cycling reactions consist of an initial denaturation (5 min at 80°C) followed by 35 cycles of denaturation (45 s at 94°C), annealing (45 s at 60°C), and extension (90 s at 72°C), with a single final extension (10 min at 72°C).

20 µl of the PCR products are purified using the QIAquick PCR Purification Kit (Qiagen, Hilden, Germany) and sequenced using the ABI Prism BigDye Terminator v3.1 Ready Reaction Cycle Sequencing Kit (Applied Biosystems, Darmstadt, Germany). The sequencing reaction contains LiChrosolv, 0.5 µl premix and 2 µl ABI 5x Sequencing Buffer from the kit, 10 pmol sequencing primer, and 2 µl cleaned PCR product in a total volume of 10 µl. The amplification primers are used for sequencing with 25 cycles of denaturation (96°C, 10 s) and extension (60°C, 4 min) at a thermal ramping of 1 ºC/s.

The sequencing products are purified with DyeEx 2.0 Spin Kit (Qiagen) and then prepared for running on the ABI 3100 Avant Genetic Analyzer in accordance with the instructions of the manufacturer (Applied Biosystems).

Type assignment is done after uploading and CQ of sequence data on the RIDOM StaphType spa server.

Annex 6 – Erasme Hospital

**Compliance monitoring procedure for standard and additional precautions**

Staff compliance with standard and additional precautions will be measured by direct observation performed by dedicated nurses (infection control team).

Fifty observations will be collected per ward and trial period, including 25 observations concerning patients under MRSA isolation precautions and 25 patients without additional isolation precautions (standard precautions). Compliance with standard precautions will limit the observation to appropriate use of hand alcoholic disinfection (using Hibiguard)

Periods of observation will be from 7.30 AM to 4:00 PM during week days.

Observation will be made by inspecting staff behaviour in the hallway before entry to the isolation room (where disposable personal protective equipment is made available) and inside the patient room (to determine the nature of the care activity and the need for additional precautions).

**Compliance rate calcualtion:**

Compliance rate with MRSA additional isolation precautions is defined as the ratio:

N° of observed situations in which gloves, gowns and, if requested, mask is used by HCW, divided by:

N° of observed situations requiring additional precautions (total denominator= 25 contacts /ward/period)

Compliance rate with standard precautions is defined as the ratio:

N° of observed situations in which alcohol hand disinfection is performed by HCW, divided by:

N° of observed situations requiring hand disinfection (total =25 contacts/ward/period)

**Best practice standard**:

Situations requiring MRSA additional isolation precautions are listed in annex 3.

For non-isolated , hospitalised patients requiring standard precautions the following situations will be included as appropriate opportunities for hand disinfection:

- before and after any direct contact with a patient’s intact skin
- before inserting indwelling urinary catheters, peripheral vascular catheter or other invasive devices that do not require a surgical procedure
- after contact with a patient’s body fluids or excretions, mucous membranes, non intact skin or wound dressings if hands are not visibly soiled (and after gloves removal)
- after contact with inanimate objects (including medical equipment) in the immediate vicinity of the patient

Appendix 7 . Patient information sheet (Fr- Nl)

**Information aux patients hospitalisés : étude du dépistage rapide du portage des staphylocoques dorés résistant à la méticilline (MRSA)**

Madame, Monsieur,

Conformément aux directives des autorités de santé, des mesures d’hygiène renforcées sont appliquées dans notre hopital pour réduire autant que possible le risque d’infection liée aux soins par les staphylocoques dorés résistant à la méticilline et à d’autres antibiotiques (MRSA). Pour cette raison, les personnes à risque d’être porteuses ce ces germes bénéficient d’analyse de laboratoire sur frottis du nez et de la peau pour dépister le portage de MRSA et adapter les soins aux personne porteuses. Ces adaptations sont les suivantes : (1) prescription si nécessaire de traitement pour éliminer le portage et/ou traiter efficacement d’éventuelles infections ; (2) mesures d’hygiène supplémentaires pour éviter la contagion à d’autre patients hospitalisés, càd séjour en chambre privée pour raison médicale (sans supplément de frais) et port par le personnel de soins de blouse en papier, masque et gants en plastique.

Nous vous informons que durant votre séjour pour soins à l’hopital, le laboratoire étudie de nouveaux tests plus rapides pour détecter le portage du MRSA sur les prélèvements ordinaires pour permettre une prise en charge adaptée plus rapide. Afin de mesurer l’utilité du test rapide sur l’efficacité des mesures d’hygiène ordinaires décrites ci-dessus, des résultats statistiques pour l’ensemble des patients hospitalisés des services inclus dans l’étude seront utilisés. Le protection habituelle de toutes les données médicales enregistrées pour cette étude est garantie. Cette étude ne comporte aucun risque pour votre bien-être car elle ne nécessite pas de prélèvements douloureux et ne modifie pas les traitements habituels.

Si vous souhaitez une information plus complète sur cette étude, le médecin chef de service et le médecin hygiéniste hospitalier sont à votre disposition.

**Inlichting voor de in het ziekenhuis opgenomen patiënten : studie van de snelle opsporing van het dragen van de meticilline-resistent stafylokokken (MRSA).**

Mevrouw, Mijnheer,

Overeenkomstig de richtlijnen van de gezondheidsinstanties, worden er in ons ziekenhuis verhoogde gezondheidsmaatregelen getroffen teneinde zo veel mogelijk het besmettinsgevaar te verminderen verbonden aan verzorging door meticilline-resistent stafylokokken (MRSA).

Om deze reden kunnen personen die het risiko lopen kiemdrager te zijn, een laboratoriumonderzoek ondergaan door een uitstrijkje van de neus en de huid om het dragen van het MRSA op te sporen en de zorgen aan de dragers ervan aan te passen. Deze aanpassingen zijn de volgende : (1) het voorschrijven, indien nodig, van de behandeling om het dragen van de kiemen te vernietigen en/of mogelijke infecties op een efficiënte manier te behandelen ; (2) bijkomende gezondheidsmaatregelen om besmetting van andere gehospitaliseerde patiënten te vermijden, d.w.z. verblijf in een eenpersoonskamer om medische redenen (zonder bijkomende kosten) en een papieren bloes, masker en plastic handschoenen door het verpleegend personeel.

Wij wensen u mede te delen dat tijdens uw verblijf voor verzorging in het ziekenhuis, het laboratorium nieuwe, snellere testen bestudeert om het dragenschap van het MRSA op te sporen op gewone stalen om zodoende een snellere, aangepaste verzorging te kunnen verstrekken. Teneinde het nut van de snelle test te meten op de doeltreffendheid van de gebruikelijke gezondheidsmaatregelen zoals hierboven beschreven, worden statistische resultaten gebruikt voor het geheel van de gehospitaliseerde patiënten van de in de studie opgenomen diensten. De gebruikelijke beveiliging van alle medische voor deze studie geregistrerde gegevens is gewaarborgd. Deze studie bevat geen enkel risiko voor uw welzijn daar zij geen pijnlijke onderzoeken vereist en uw gewone behandeling in niets verandert.

Mocht u meer gedetailleerde inlichtingen wensen te bekomen aangaande deze studie, dan kan u zich wenden tot de Geneesheer Diensthoofd van uw afdeling of de Geneesheer Hygiënist van het ziekenhuis.
