# Supplementary material for: Impact of Rapid Molecular Screening at Hospital Admission on Nosocomial Transmission of Methicillin-Resistant Staphylococcus aureus: Cluster Randomised Trial
Source: PLoS One. 2014 May 16;9(5):e96310. doi: 10.1371/journal.pone.0096310 (PMC4023928; doi:10.1371/journal.pone.0096310)
Supplement: File S1 — Ethics approval document. (PDF) [file pone.0096310.s003.pdf]

CLINIQUES UNIVERSITAIRES DE BRUXELLES
UNIVERSITE LIBRE
DE BRUXELLES
HOPITAL ERASME

ULB

ROUTE DE LENNIK 808 - B-1070 BRUXELLES

Brussels, the September 16, 2008

COMITE D'ETHIQUE
Agréation: N°OM021

Pr. Marc STRUELENS
Department of Bacteriology &Virology
CUB Hôpital Erasme
Route de Lennik 808
B-1070Bruxelles

Secrétariat: Tél.: 32 2.555.37.07
Fax: 32.2.555.46.20
E-mail: ethique.hopitalerasme@ulb.ac.be

Dear Pr. STRUELENS,

Please find further on the answer of the Ethics Committee concerning the below mentioned study.

ETHICS COMMITTEE APPROVAL

We, undersigned,	Pr. A. HERCHUELZ & Mr. G. NISET
Chairman & Secretary of	Ethics Committee Erasme Hospital<br>808, route de Lennik<br>B-1070 Brussels, Belgium<br>N° of agreation by "Ordre des Médecins": OM021

confirm that the documents submitted for approval were initially examined during the meeting held on 19/08/2008 (dd/mm/yyyy).

After collecting the opinion of the non-leading ethics committees and after examination of the answers to the comments and of the new version of the information sheet and consent form received the 15/09/2008, the Ethics Committee gave its agreement on 16/09/2008 to the following documents:

- Protocol (Our Ref.: P2008/201) entitled: *"Two-center intervention study to evaluate the impact of rapid molecular screening on nosocomial transmission of Methicillin-resistant Staphylococcus aureus (MRSA)."* (Version Final protocol V8, dated July 30, 2008)
- French Patient Information Sheet: Version 2
- Dutch Patient Information Sheet: Version 2
- Investigator's Curriculum Vitae
- Ethics Committees Application form signed the 7/31/2008
- Table "Data to be collected"

The list of the names and qualifications of the members of the Ethics Committee present at the meeting and the list of the Ethics Committees concerned (in case of multi-centre study) are added in appendix.

The Committee stresses that it is the responsibility of the promoter to guarantee the conformity of Patient Information sheet in French and Dutch. We consider also the promoter's responsibility to provide to all the peripherals ethics committees the final French and Dutch versions of the patient information sheet (adapted, if needed with the name of the local investigator, the name of the institution,...) approved in the present letter.

We hereby confirm that this Ethics Committee is organized and operates according to ICH GCP and the applicable law and regulations.

Mr. G. NISET
Secretary

Pr. A. HERCHUELZ
Chairman

CLINIQUES UNIVERSITAIRES DE BRUXELLES
UNIVERSITE LIBRE
DE BRUXELLES
HOPITAL ERASME

ROUTE DE LENNIK 808 - B-1070 BRUXELLES

COMITE D'ETHIQUE
Agréation: N°OM021

List of Members present at the meeting of 19/08/2008 (dd/mm/yyyy)

Secrétariat: Tél.: 32.2.555.37.07
Fax: 32.2.555.46.20
E-mail: comite.ethique@erasme.ulb.ac.be

Président :
Pr. A. Herchuelz
Vice-Présidents :
Pr. S. Goldman
Dr. M. Remmelink
Secrétaire :
Mr. G. Nisel

Membres :
Dr. X. CATTEAU
Dr. E. DEMANET
Dr. P. DEMETTER
Dr. S. DE BREUCKER
Mr. P. FISCHBACH
Me. B. FONTEYN
Me. P.-A. FORIERS
Mme. N. GAMMAR
Dr. S. GOLDMAN
Dr. A. HERCHUELZ
Dr. H. LOUIS
Dr. M. MANTO
Dr. J. MARIN
Dr. F. MASCART
Mr. M. MAYER
Mme. A. NELIS
Mr. G. NISET
Mme. A. OCMANT
Dr. M. REMMELINK
Dr. J. ROUBY
Mme. C. SAUVAGE
Dr. P. THIBAUT
Dr. J.L. VACHIERY
Dr. C. VERBEURGT

■ = present
□ = absent
E = Absent but excused

■	Dr. X. CATTEAU, Pathologist, Medical Doctor, Male
■	Dr. E. DEMANET, General Practitioner, Medical Doctor, Male
E	Dr. P. DEMETTER, Pathologist, Medical Doctor, Male
E	Dr. S. DE BREUCKER, Geriatrist, Medical Doctor, Female
■	Mr. P. FISCHBACH, Psychologist, Male
E	Me. B. FONTEYN, Lawyer, Male
■	Me. P.-A. FORIERS, Lawyer, Male
■	Mme. N. GAMMAR, Assistant Nursing Director, Nurse, Female
E	Pr. S. GOLDMAN, Neurologist, Medical Doctor, Male
■	Pr. A. HERCHUELZ, Pharmacologist, Medical Doctor, Male
E	Dr. H. LOUIS, Gastroenterologist, Medical Doctor, Male
E	Dr. M. MANTO, Neurologist, Medical Doctor, Male
E	Dr. J. MARIN, General Practitioner, Medical Doctor, Male
■	Pr. F. MASCART, Immunobiologist, Medical Doctor, Female
■	Mr. M. MAYER, Lay member, Moral Counsel, Male
E	Mme. A. NELIS, Assistant Nursing Director, Nurse, Female
■	Mr. G. NISET, Ethics Committee Secretary, Male
■	Mme. A. OCMANT, Pharmacist, Female
■	Dr. M. REMMELINK, Pathologist, Medical Doctor, Female
■	Dr. J. ROUBY, General Practitioner, Medical Doctor, Male
E	Mme C. SAUVAGE, Physiotherapist, Female
E	Dr. P. THIBAUT, General Practitioner, Medical Doctor, Male
■	Dr. J.-L. VACHIERY, Cardiologist, Medical Doctor, Male
■	Dr. C. VERBEURGT, Medical Doctor, Male

Edited August 19, 2008
